# Softness‐Aided Mild Hyperthermia Boosts Stiff Nanomedicine by Regulating Tumor Mechanics

**DOI:** 10.1002/advs.202306730

**Published:** 2024-05-05

**Authors:** Zheng Li, Yabo Zhu, Zhijie Zhang, Huimin Wang, Chong Wang, Chen Xu, Shiyou Li, Shuya Zhang, Xiangliang Yang, Zifu Li

**Affiliations:** ^1^ Department of Nanomedicine and Biopharmaceuticals College of Life Science and Technology Huazhong University of Science and Technology Wuhan 430074 P. R. China; ^2^ National Engineering Research Center for Nanomedicine Huazhong University of Science and Technology Wuhan 430074 P. R. China; ^3^ Key Laboratory of Molecular Biophysics of Ministry of Education Huazhong University of Science and Technology Wuhan 430074 P. R. China; ^4^ Hubei Key Laboratory of Bioinorganic Chemistry and Materia Medical Huazhong University of Science and Technology Wuhan 430074 P. R. China; ^5^ Hubei Engineering Research Center for Biomaterials and Medical Protective Materials Huazhong University of Science and Technology Wuhan 430074 P. R. China; ^6^ Hubei Bioinformatics and Molecular Imaging Key Laboratory Huazhong University of Science and Technology Wuhan 430074 P. R. China

**Keywords:** mild‐hyperthermia photothermal therapy, nanogel stiffness, tumor mechanical microenvironment, tumor penetration, tumor therapy

## Abstract

Aberrant tumor mechanical microenvironment (TMME), featured with overactivated cancer‐associated fibroblasts (CAFs) and excessive extracellular matrix (ECM), severely restricts penetration and accumulation of cancer nanomedicines, while mild‐hyperthermia photothermal therapy (mild‐PTT) has been developed to modulate TMME. However, photothermal agents also encounter the barriers established by TMME, manifesting in limited penetration and heterogeneous distribution across tumor tissues and ending with attenuated efficiency in TMME regulation. Herein, it is leveraged indocyanine green (ICG)‐loaded soft nanogels with outstanding deformability, for efficient tumor penetration and uniform distribution, in combination with mild‐PTT to achieve potent TMME regulation by inhibiting CAFs and degrading ECM. As a result, doxorubicin (DOX)‐loaded stiff nanogels gain greater benefits in tumor penetration and antitumor efficacy than soft counterparts from softness‐mediated mild‐PTT. This study reveals the crucial role of nanomedicine mechanical properties in tumor distribution and provides a novel strategy for overcoming the barriers of solid tumors with soft deformable nanogels.

## Graphical abstract

Soft and stiff nanogels are prepared to deliver ICG for mild‐PTT. ICG‐loaded soft nanogels present more advantages in CAFs depletion and ECM degradation relying on deeper penetration and more uniform distribution in tumor tissues compared to the stiff counterparts. After mild‐PTT, DOX‐loaded stiff nanogels obtain greater promotion in tumor accumulation, deep penetration and antitumor efficacy, than the soft counterparts.

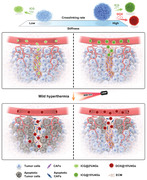

## 1 Introduction

Nanomedicine has been intensively pursued for tumor targeting delivery of small molecular drugs via the enhanced permeability and retention (EPR) effect for decades.^[^ ^1^ ^]^ Although many physicochemical properties of nanomedicines such as size, shape, surface charge, chemical composition, surface modification and so on, have been investigated and optimized for prolonged blood circulation and efficient tumor accumulation,^[^ ^2^ ^]^ they are still facing impediment from abnormal tumor mechanical microenvironment (TMME).^[^ ^3^ ^]^ Overactivated cancer‐associated fibroblasts (CAFs) secreted superabundant matrix proteins such as collagen and fibronectin, which are the major components of dense extracellular matrix (ECM),^[^ ^3^, ^4^ ^]^ altogether establishing barriers for drug delivery. In particular, narrow gaps among ECM hinder nanomedicine from penetrating away from blood vessels to tumor interior, leading to heterogeneous distribution around blood vessels and limited antitumor efficacy.^[^ ^5^ ^]^ Therefore, depletion of CAFs and degradation of ECM were key approaches to removal of obstacles for effective tumor penetration and antitumor efficacy.

To overcome the hindrance from abnormal TMME, many strategies have been developed.^[^ ^6^ ^]^ As an example, transforming growth factor‐β (TGF‐β) inhibitor LY2157299 could decrease collagen deposition by suppressing TGF‐β signaling pathway and facilitate photodynamic therapy of hydroxyethyl starch‐chlorin e6 conjugate self‐assembled nanoparticles via improving tumor accumulation and penetration.^[^ ^6c^ ^]^ Hyperbaric oxygen regulates tumor mechanics and promotes antitumor effects of commercialized nanomedicine Doxil, Abraxane and programmed cell death protein 1 antibody.^[^ ^6^, ^7^ ^]^ Photothermal therapy (PTT) has also been leveraged to modulate TMME.^[^ ^8^ ^]^ In 1990s, higher‐power laser has been introduced to produce large amount of thermal energy and degrade collagen of skin in piglet models.^[^ ^9^ ^]^ Robust PTT induced by bioinspired lipoprotein can disrupt stroma cells and ECM components to remodel tumor stroma microenvironments and endow accessibility of second bioinspired lipoprotein to tumor cells.^[^ ^10^ ^]^ In consideration of locally‐activated and manually‐controlled operation, PTT presents enormous potential for TMME improvement.

As a key parameter for PTT, although high temperature can induce excellent tumor ablation and contribute to encouraging achievements in cancer therapy,^[^ ^11^ ^]^ superfluous thermal conduction to surrounding normal tissues results in unexpected thermal damages and inflammatory reactions at the same time.^[^ ^12^ ^]^ So, mild‐PTT with temperature lower than 45 °C becomes a more eligible option to avoid thermal damages of normal tissues,^[^ ^13^ ^]^ which has been demonstrated to be effective in remodeling TMME.^[^ ^14^ ^]^ For instance, multifunctional iron oxide nanoflowers decorated with gold nanoparticles as efficient nanoheaters for mild‐PTT can significantly deplete CAFs and normalize tumor mechanics.^[^ ^14a^ ^]^ Locally mild‐PTT by olaparib and doxorubicin (DOX) co‐loaded PEGylated mesoporous polydopamine nanoparticles on tumor tissues can repress CAFs and decrease ECM via normalizing hypoxia.^[^ ^14b^ ^]^ Interestingly, it is reported that both thermal ablation and mild hyperthermia can transiently and reversibly increase tumor stiffness after laser exposure, while tumor stiffness of mild hyperthermia decreases to a lower level than thermal ablation 24 h post treatments.^[^ ^14c^ ^]^ However, photothermal agents, either small molecules or nanotherapeutics, also encounter the obstacles from the dense ECM, ending with inhomogeneous distribution and poor penetration. The function of mild‐PTT‐mediated ECM degradation is critically restricted to local tumor tissues.^[^ ^3^, ^15^ ^]^ To solve this issue, physicochemical properties of nanoparticles as nanocarriers for photothermal agents should be optimized.

Mechanical properties of nanoparticles, as a rather new parameters of nanomedicine, play an important role in drug delivery, especially tumor penetration.^[^ ^16^ ^]^ For example, due to the excellent deformability, soft DOX‐loaded 3D microparticles can squeeze through dense ECM among tumor cells and exhibit better permeability in tumor tissues than stiff counterparts.^[^ ^17^ ^]^ In our previous work, soft DOX‐loaded nanogels with cross‐linking rate of 2% (DOX@2%NGs) with low network density also demonstrated more encouraging tumor penetration and antitumor efficacy.^[^ ^18^ ^]^ As a widely‐used nanocarrier with excellent stability, hydrophilicity and biocompatibility, nanogel has been developed for multifarious applications in drug delivery, including photothermal agent indocyanine green (ICG) and antitumor agent DOX.^[^ ^19^ ^]^ Owe to the unique network structure and easily‐controlled deformability, nanogels are widely used as model nanoparticles to investigate mechanical properties of nanocarriers and the interactions with cells or organs.^[^ ^18^, ^20^ ^]^ Herein, *N*‐isopropylmethacrylamide (NIPMAM) was utilized as monomer instead of N‐isopropylacrylamide (NIPAM), because of higher volume phase transition temperature (VPTT) and extra deformability under physiological conditions.^[^ ^21^ ^]^

**Figure 1:**
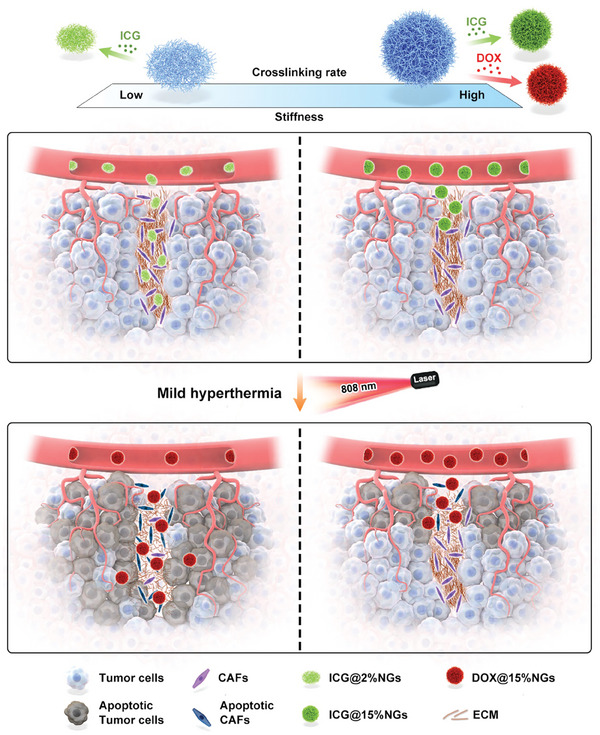
Schematic illustration of softness‐aided mild hyperthermia boosts stiff nanomedicine by regulating tumor mechanics. ICG‐loaded soft nanogels (ICG@2%NGs) as photothermal agents with outstanding deformability can achieve efficient tumor penetration and uniform distribution. After irradiation by 808 nm laser for mild‐PTT, ICG‐loaded soft nanogels potently inhibit CAFs and degrade ECM. Afterwards, DOX‐loaded stiff nanogels (DOX@15%NGs) gain greater benefits in tumor penetration and antitumor efficacy from soft nanogels‐mediated mild‐PTT.

In this work, we prepared two kinds of poly(*N*‐isopropylmethacrylamide‐disulfide bond‐methacrylic acid) (P(NIPMAM‐ss‐MAA)) nanogels with varied stiffness containing soft 2%NGs and stiff 15%NGs. After loading photothermal agent ICG and irradiation by near infrared laser with mild temperature below 43 °C, soft ICG@2%NGs exhibited superiority in CAFs inhibition and ECM degradation at similar temperature, relying on better permeability and distribution than stiff ICG@15%NGs. Thanks to ICG@2%NGs‐mediated TMME improvement, tumor accumulation, tumor penetration and antitumor efficacy of DOX‐loaded nanogels gained benefits, among which stiff DOX@15%NGs profited more than soft counterparts. Mechanistically, it was revealed that the penetration of nanomedicine could be hardly augmented by photothermal agents with similar stiffness or permeability. Because the photothermal agents could only achieve close penetration depth to the nanomedicine, failing to degrade deeper ECM or enhance further penetration of the nanomedicine. Instead, these photothermal agents could degrade ECM around the nanomedicine and increase enrichment of the nanomedicine in such area. Solely increasing nanomedicine enrichment at superficial tumor tissue made limited contribution to nanomedicine antitumor efficacy because no deeper tumor cells were eliminated. In contrast, photothermal agents with lower stiffness or stronger permeability than the nanomedicine could degrade deeper ECM and benefit for nanomedicine penetration, leading to more sufficient tumor cell elimination and antitumor effect. In other words, to remove the obstacles of aberrant TMME and promote nanomedicines deep penetration, softer photothermal agents should be applied first (**Figure** **1**). In clinical applications, stiffness of nanomedicine enhancers like photothermal agents should be coordinated and optimized to achieve robust enhancement in tumor penetration and antitumor efficacy of nanomedicines.

## 2 Results and Discussion

### 2.1 Characterization of ICG‐Loaded P(NIPMAM‐ss‐MAA) Nanogels with Varied Stiffness

To investigate how the nanogel mechanical properties affected delivery of photothermal agents, we synthesized two kinds of P(NIPMAM‐ss‐MAA) nanogels with distinctive stiffness via emulsion polymerization.^[^ ^18^ ^]^ The P(NIPMAM‐ss‐MAA) nanogels contained temperature‐responsive monomer NIPMAM, which was introduced to construct network structure of nanogels for outstanding deformability at 37 °C, and pH‐responsive monomer MAA, which was used to provide additional hydrophilicity for stability of nanogels and negative surface charge for drug loading via electrostatic interaction. The molar ratio between crosslinker N,N’‐bis(acryloyl)cystamine (BAC) and NIPMAM was regulated to obtain soft nanogels with cross‐linking rate of 2% (2%NGs) and stiff nanogels with cross‐linking rate of 15% (15%NGs). In addition, the disulfide bond of BAC rendered nanogels glutathione‐responsive (GSH‐responsive) and biodegradable properties. The amount of surfactant sodium dodecyl sulfate (SDS) was precisely controlled to ensure similar size distribution of nanogels with different stiffness under physiological condition. At the same time, Rhodamine B (RhB)‐labeled nanogels with different stiffness were also prepared including RhB@2%NGs and RhB@15%NGs. Ulteriorly, hydrophilic chemotherapy DOX·HCl was loaded onto nanogels via electrostatic interaction and photothermal agent ICG was further loaded via π–π interaction with DOX, to obtain ICG@2%NGs and ICG@15%NGs.

**Figure 2:**
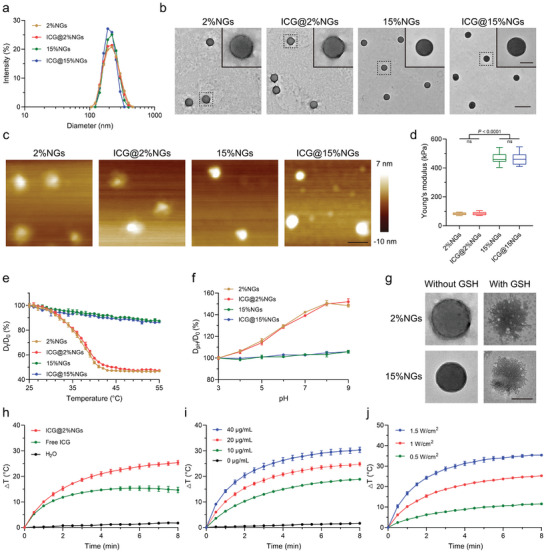
Characterization of ICG‐loaded P(NIPMAM‐ss‐MAA) nanogels with varied stiffness. a) Size distribution of ICG‐loaded nanogels with varied stiffness in PBS at 37 °C. b) TEM images of ICG‐loaded nanogels with varied stiffness. Scale bar = 500 nm in low magnification and Scale bar = 100 nm in high magnification. c) AFM images of ICG‐loaded nanogels with varied stiffness in air phase. Scale bar = 200 nm. d) Young‘s modulus of ICG‐loaded nanogels with varied stiffness in liquid phase. Box plots indicate median (middle line), 25th, 75th percentile (box) and minimum and maximum (whiskers), (*n* = 20 independent replicates). e) Temperature‐responsiveness of ICG‐loaded nanogels with varied stiffness in H_2_O. Data are presented as mean values ± SD (*n* = 3 independent replicates). f) pH‐responsiveness of ICG‐loaded nanogels with varied stiffness in H_2_O. Data are presented as mean values ± SD (*n* = 3 independent replicates). g) TEM images of 2%NGs and 15%NGs incubated with or without GSH for 24 h. Scale bar = 100 nm. h) Photothermal properties of free ICG and ICG@2%NGs in H_2_O with ICG concentration of 20 µg/mL and 808 nm laser power of 1 W/cm^2^. Data are presented as mean values ± SD (*n* = 3 independent replicates). i) Concentration‐dependent photothermal properties of ICG@2%NGs in H_2_O with 808 nm laser power of 1 W cm^−2^. Data are presented as mean values ± SD (*n* = 3 independent replicates). j) Power‐dependent photothermal properties of ICG@2%NGs in H_2_O with ICG concentration of 20 µg mL^−1^. Data are presented as mean values ± SD (*n* = 3 independent replicates). Statistical significance was calculated by one‐way ANOVA.

The size distribution results confirmed that the hydrodynamic diameter of 2%NGs and 15%NGs were both ≈220 nm in PBS at 37 °C which simulated physiological condition (**Figure** **2**a). Zeta potential of 2%NGs and 15%NGs was −15.00 ± 0.17 mV and −22.73 ± 0.15 mV, respectively (Figure S1, Supporting Information). Monodispersed spherical morphologies of nanogels could be observed through transmission electron microscopy (TEM) and atomic force microscopy (AFM) images (Figure 2b,c). However, dehydration during sample preparation inevitably caused shrinkage of nanogel network structure and discrepancy in diameter between 2%NGs and 15%NGs. To investigate whether the nanogel stiffness was positively correlated with cross‐linking rate, Young's modulus was measured, of which the soft 2%NGs was 82.92 ± 7.70 kPa while the stiff 15%NGs was 468.95 ± 40.74 kPa (Figure 2d). On the other hand, deformability was another indicator to evaluate the mechanical properties of nanogels.^[^ ^22^ ^]^ The temperature‐responsiveness and pH‐responsiveness of nanogels demonstrated that soft 2%NGs presented stronger shrinkage or swelling with increase of temperature or pH, compared to stiff 15%NGs (Figure 2e,f; Figure S2, Supporting Information). After incubation with 10 mmol L^−1^ GSH for 24 h, obvious destruction of nanogel structure could be observed in TEM images (Figure 2g). Besides, physicochemical properties of ICG‐loaded nanogels kept consistent with nanogels without loading ICG (Figure 2a–g; Figure S1 and S2, Supporting Information).

Although ICG was hydrophilic and has been widely used for bioimaging in clinical settings, nanocarriers could further enhance photostability and tumor accumulation of ICG.^[^ ^23^ ^]^ The UV–vis absorption spectra revealed that the maximum absorption wavelength (*λ*_max_) of ICG in dimethyl sulfoxide (DMSO) was 795.5 nm and the *λ*_max_ of ICG in H_2_O was 780 nm. However, the *λ*_max_ of ICG@2%NGs in H_2_O was 784.5 nm, which meant nanogels as nanocarriers contributed to *λ*_max_ shifting toward longer wavelength in H_2_O for better optical penetration and stronger absorption of 808 nm laser (Figure S3, Supporting Information). Furthermore, ICG@2%NGs exhibited more efficient photothermal properties, of which the temperature increment of 25.37 ± 0.85 °C after irradiation by 808 nm laser with power of 1 W cm^−2^ for 8 min in H_2_O whereas only increment of 14.63 ± 1.07 °C was detected for free ICG (Figure 2h; Figure S4, Supporting Information). In addition, ICG@2%NGs still possessed superior photothermal capabilities after irradiation by 808 nm laser for several rounds, and the UV–vis absorption spectra also demonstrated better photostability of ICG@2%NGs than free ICG in H_2_O after irradiation for 3 min (Figure S5, Supporting Information). Afterwards, concentration‐dependent (Figure 2i; Figure S6, Supporting Information) and power‐dependent (Figure 2j; Figure S7, Supporting Information) photothermal properties of ICG@2%NGs were evaluated. Faster and higher temperature rise was detected with increasing of ICG concentration and power of 808 nm laser.

To sum up, nanogels with similar physicochemical properties but different stiffness, were synthesized by regulating cross‐linking rate, including soft 2%NGs and stiff 15%NGs. After loading DOX and ICG, soft ICG@2%NGs and stiff ICG@15%NGs were obtained correspondingly, and photothermal efficiency along with photostability in H_2_O was obviously increased, giving support for in vitro and in vivo applications.

### 2.2 Mild Hyperthermia Enhances Nanomedicine Tumor Accumulation by Increased Blood Perfusion and Complete Blood Vasculature

Although hyperthermia leads to excellent tumor ablation, heterogeneous distribution of photothermal agents results in recurrence of untreated tumors post PTT.^[^ ^13^, ^15^, ^24^ ^]^ It has been reported that mild hyperthermia can increase the blood flow of tumor blood vessels,^[^ ^25^ ^]^ whereas hyperthermia will cause hemorrhage and stasis of tumor vessels.^[^ ^26^ ^]^ Furthermore, in consideration of the requirements for subsequent drug administration post TMME regulation, maintaining structure integrity of tumor blood vessels was essential.

**Figure 3:**
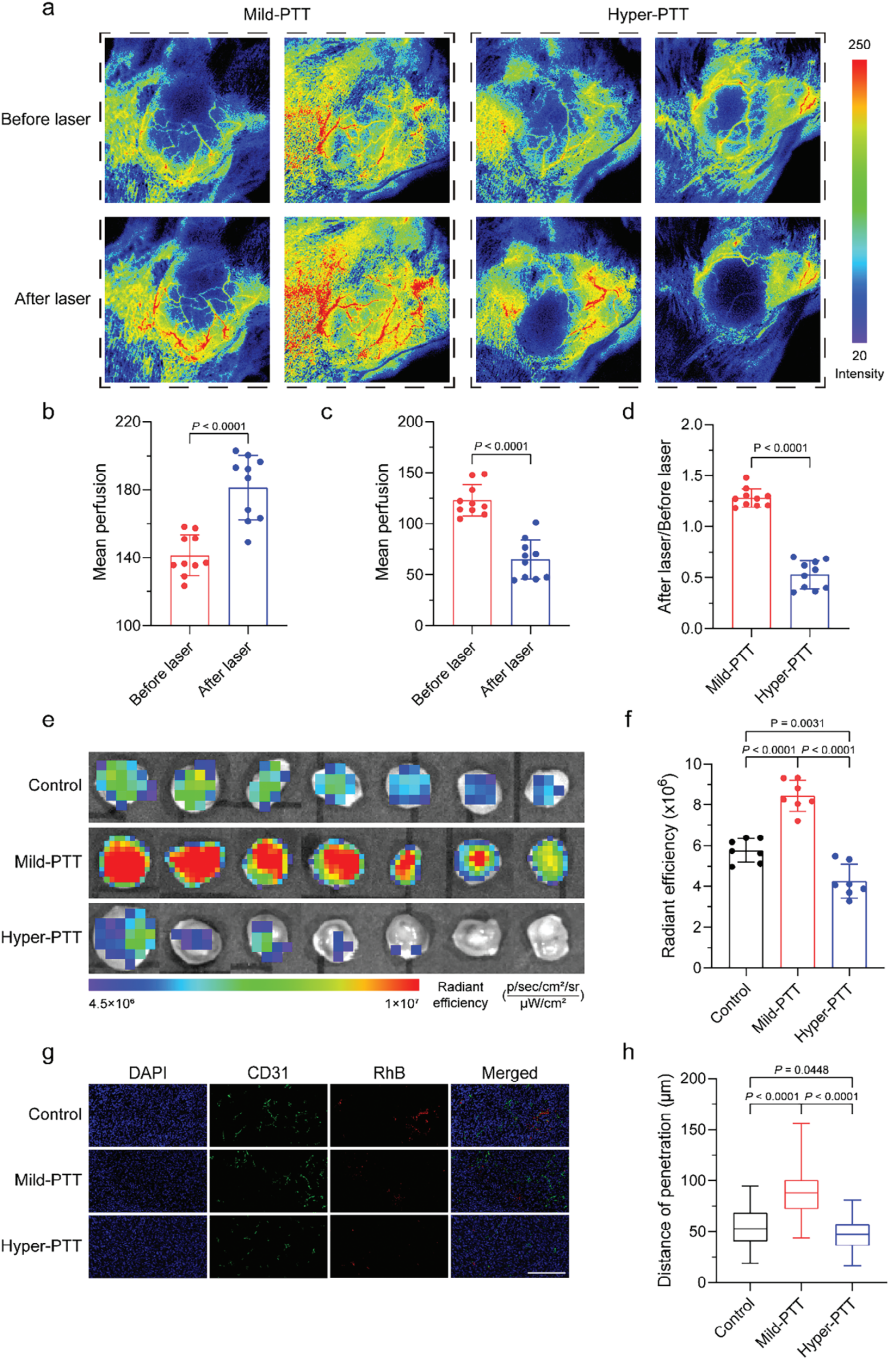
Mild hyperthermia enhances nanomedicine tumor accumulation by increased blood perfusion and complete blood vasculature. a) Blood perfusion after ICG@2%NGs‐mediated mild‐PTT or hyper‐PTT. b) Semi‐quantification of blood perfusion increment after mild‐PTT. Data are presented as mean values ± SD (*n* = 10 independent replicates). c) Semi‐quantification of blood perfusion decrement after hyper‐PTT. Data are presented as mean values ± SD (*n* = 10 independent replicates). d) Relative variation of blood perfusion after mild‐PTT or hyper‐PTT. Data are presented as mean values ± SD (*n* = 10 independent replicates). e) Ex vivo fluorescent images of tumor accumulation of RhB@15%NGs after ICG@2%NGs‐mediated mild‐PTT or hyper‐PTT. f) Semi‐quantification of tumor accumulation of RhB@15%NGs in e). Data are presented as mean values ± SD (*n* = 7 biological independent replicates). g) CD31 staining of tumors in e). Blue: nuclei of tumor cells. Green: blood vessels. Red: RhB@15%NGs. Scale bar = 200 µm. h) Distance from RhB@15%NGs to the nearest blood vessel in g). Box plots indicate median (middle line), 25th, 75th percentile (box) and minimum and maximum (whiskers) (*n* = 80 independent replicates). Statistical significance of f,h) was calculated by one‐way ANOVA. Statistical significance of b,c,d) was calculated by unpaired two‐sided Student's t‐test.

Herein, ICG@2%NGs was used as photothermal agent via intravenous (i.v.) injection and irradiated by 808 nm laser. The laser area was 0.75 cm^2^ and the distance over tumors was 2 cm. The laser irradiation was started with the power of 1 W cm^−2^ and the tumor temperature was monitored with infrared thermal imager. For mild‐PTT group, when the tumor temperature reached 43 °C, the laser power was turned down and adjusted manually to maintain tumor temperature below 43 °C. As for hyper‐PTT group, tumors were continuously irradiated by laser with power of 1 W cm^−2^ and the tumor temperature would increase over 50 °C. The whole irradiation process retained for 10 min. The results revealed that after mild‐PTT below 43 °C for 10 min, the blood perfusion in tumor tissue was significantly enhanced (**Figure** **3**a,b). In striking contrast, the blood perfusion could hardly be detected in core area of laser irradiation with the highest temperature over 50 °C, while larger amount of blood perfusion could still be observed at tumor periphery with relatively milder temperature (Figure 3a,c). Relative variation of blood perfusion was also calculated (Figure 3d). Accordingly, we reasoned that hyper‐PTT might cause serious damage to blood vessels and the blood flow was intercepted. Theoretically, blood perfusion expansion could increase the amount of nanomedicine accumulated at tumor site and promote drug delivery to tumor cells.^[^ ^7^, ^27^ ^]^ The ex vivo biodistribution demonstrated that tumor accumulation of RhB@15%NGs after mild‐PTT was much higher than the control group without laser irradiation. Consistent with our prediction, tumor accumulation of RhB@15%NGs after hyper‐PTT treatment was obviously reduced (Figure 3e,f). The fluorescent images of anti‐CD31 antibody‐labeled blood vessels and RhB‐labeled nanogels demonstrated that mild‐PTT enhanced the penetration of 15%NGs from 53.70 ± 18.84 to 87.43 ± 21.58 µm, whereas hyper‐PTT made no difference (Figure 3g,h) relative to control. Besides, mild‐PTT commendably maintained the tumor blood vessel vasculature and the length of blood vessels was close to control group (Figure S8, Supporting Information). In hyper‐PTT group, blood vessels were severely damaged and fractured with length shorter than mild‐PTT. These results demonstrated mild‐PTT augmented nanomedicine tumor accumulation by enhancing blood perfusion and maintaining tumor blood vasculature while hyper‐PTT deteriorated nanomedicine tumor accumulation via disrupting structure integrity and function of tumor blood vessels.

Furthermore, we took commercialized nanomedicine Doxil as an example to explore whether mild‐PTT‐enhanced blood perfusion could indeed increase antitumor efficacy of nanomedicines. 4T1 subcutaneous tumor models were established and randomly divided into five groups including control (G1), ICG@2%NGs (G2), ICG@2%NGs (laser) (G3), ICG@2%NGs + Doxil (G4), ICG@2%NGs (laser) + Doxil (G5). Among these groups, ICG@2%NGs as photothermal agent was i.v. injected preferentially, then Doxil was administered as therapeutic agent followed by 808 nm laser irradiation with manually‐controlled temperature below 43 °C for 10 min. This operation was proceeded on day 1 and day 7 (Figure S9a, Supporting Information). Although ICG@2%NGs (G2) contained DOX, the small amount of DOX and low drug administration frequency could hardly induce antitumor effect. Mild‐PTT resulted in partial thermal damage in tumor, but it did not affect the overall trend of tumor growth and no significant difference could be detected between G2 and G3. Doxil exhibited relatively good antitumor efficacy with tumor volume of 335.95 ± 64.60 mm^3^ as a commercialized nanomedicine for clinical tumor therapy in G4, which was further promoted by ICG@2%NGs‐mediated mild‐PTT in G5 with tumor volume of 205.80 ± 47.48 mm^3^ (Figure S9b, Supporting Information). Consistently, tumor weight and tumor photo also confirmed that the combination of Doxil with mild‐PTT contributed to the optimal antitumor efficacy (Figure S9c,d, Supporting Information). Meanwhile, volume‐based and weight‐based tumor inhibition rate was calculated (Figure S10, Supporting Information). After i.v. injection of Doxil, the following mild‐PTT obviously increased tumor blood perfusion without disrupting tumor blood vasculature. At the same time, more Doxil reached tumor cite and penetrated deeper tumor tissue, leading to the highest tumor inhibition rate for G5 among all groups. The harvested tumors were fixed with 4% paraformaldehyde for H&E, Caspase‐3 and Ki67 staining to evaluate necrosis, apoptosis, and proliferation of tumor cells. The sparest distribution of tumor cells, the highest percentage of apoptotic cells and the lowest percentage of proliferative cells were observed in G5 (Figure S11, Supporting Information). To investigate the safety of these treatments, mice body weight was measured, major organs were harvested for H&E staining and blood was collected for blood biochemical analysis and blood routine examination. The effect of mild‐PTT on body weight was negligible. Administration of therapeutic agents slightly decreased the body weight in short term and the body weight recovered to normal level over time (Figure S9e, Supporting Information). H&E staining also proved that no toxicity was generated in major organs by such treatments (Figure S12, Supporting Information). In blood biochemical analysis and blood routine examine, the number of white blood cells (WBC) was positively corelated with tumor volume as a probable result of tumor malignancy and inflammation,^[^ ^28^ ^]^ but all the physiological indicators were still in the normal range (Figure S13, Supporting Information). So, ICG@2%NGs‐mediated mild‐PTT exhibited outstanding promotion on nanomedicine antitumor efficacy without unexpected side effects.

In summary, mild‐PTT was demonstrated to be a more appropriate treatment for maintaining blood vessel's structure integrity and transfusing subsequent therapeutic agents than traditional hyper‐PTT. Additionally, mild‐PTT significantly increased tumor accumulation and antitumor efficacy of nanomedicines (e.g., Doxil), relying on expanded blood perfusion and complete blood vasculature. In contrast, destruction of blood vessels by hyper‐PTT resulted in inaccessibility of nanomedicine to tumor tissue and insufficient tumor accumulation. As for TMME modulation, which also required for multiple rounds of drug administration, mild‐PTT became a reasonable option.

### 2.3 Soft Nanogels Achieves Deeper Penetration and More Uniform Distribution in Tumor Tissue than Stiff Counterparts

Although mild‐PTT was considered as an appropriate therapeutic of tumor mechanics regulation for subsequent drug accumulation, limited penetration and heterogeneous distribution of photothermal agents restricted TMME modulation efficiency, to a great extent.^[^ ^3^, ^15^ ^]^ Many studies demonstrated that mechanical properties of nanoparticles, especially stiffness, played an important role in overcoming dense ECM and achieving deep penetration.^[^ ^16^ ^]^ So, it was of vital significance to investigate whether soft 2%NGs also made contribution to tumor accumulation and tumor penetration of ICG so as to improve TMME regulation efficiency.

**Figure 4:**
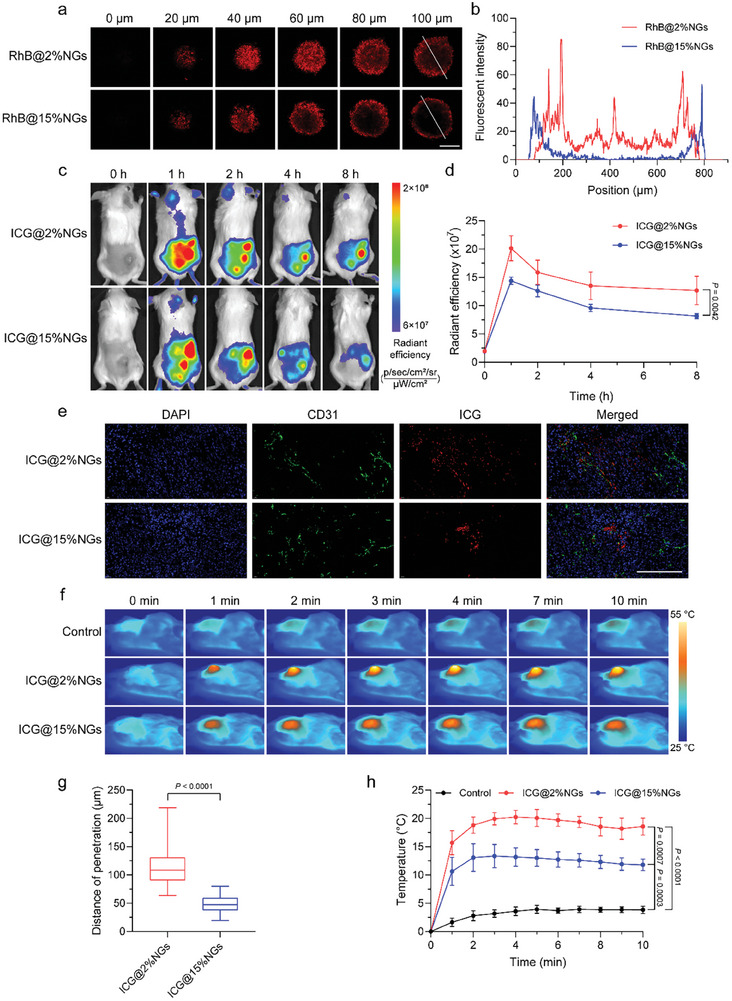
Soft nanogels achieves deeper penetration and more uniform distribution in tumor tissue than stiff counterparts. a) Permeability of Rhodamine B‐labeled nanogels with different stiffness in 3D tumor spheroids. Scale bar = 200 µm. b) Distribution of fluorescent intensity along radial direction in a). c) In vivo fluorescent images of tumor accumulation of ICG‐loaded nanogels with different stiffness. d) Semi‐quantification of tumor accumulation of ICG‐loaded nanogels in c). Data are presented as mean values ± SD (*n* = 5 biological independent replicates). e) CD31 staining of tumors. Blue: nuclei of tumor cells. Green: blood vessels. Red: ICG‐loaded nanogels. Scale bar = 200 µm. f) Infrared images of in vivo photothermal properties of ICG‐loaded nanogels with different stiffness. g) Distance from ICG‐loaded nanogels with different stiffness to the nearest blood vessel in e). Box plots indicate median (middle line), 25th, 75th percentile (box) and minimum and maximum (whiskers) (*n* = 80 independent replicates). h) In vivo photothermal properties of ICG‐loaded nanogels with different stiffness in f). Data are presented as mean values ± SD (*n* = 3 biological independent replicates). Statistical significance of h) was calculated by one‐way ANOVA. Statistical significance of d,g) was calculated by unpaired two‐sided Student's t‐test.

To investigate how stiffness affected nanogels permeability, we utilized RhB‐labeled nanogels with distinctive stiffness instead of ICG‐loaded nanogels to avoid the influence of released ICG, and used 3D tumor spheroids as in vitro tumor models. The results confirmed that soft RhB@2%NGs exhibited better permeability than stiff RhB@15%NGs (**Figure** **4**a,b). Fluorescent intensity of RhB@2%NGs could still be detected in spheroid core at the depth of 100 µm, whereas RhB@15%NGs mostly accumulated at spheroid periphery and led to stronger fluorescent intensity at surface layer. Better permeability of soft 2%NGs contributed to higher tumor accumulation of ICG@2%NGs, of which the fluorescent intensity was ≈56% higher than ICG@15%NGs (Figure 4c,d). Afterwards, fluorescent imaging of anti‐CD31 antibody‐labeled blood vessels and ICG‐loaded nanogels also demonstrated that soft ICG@2%NGs could penetrate further away from blood vessels with distance of 112.02 ± 28.68 µm; instead, stiff ICG@15%NGs only penetrated with distance of 48.98 ± 14.74 µm (Figure 4e,g). It should be mentioned that more uniform distribution of ICG@2%NGs was observed along blood vessels and across tumor tissues, leading to more extensive potential of regulating tumor mechanics. In contrast, ICG@15%NGs distributed partially close to blood vessels with high concentration, consistent with large amount of RhB@15%NGs at 3D tumor spheroid periphery, and the ability of TMME modulation might be limited. Benefiting from higher tumor accumulation and deeper tumor penetration, ICG@2%NGs exhibited much stronger photothermal ability than ICG@15%NGs (Figure 4f,h; Figure S14, Supporting Information). Even though, only mild temperature below 43 °C from macroscopic level was needed in this work to avoid unexpected thermal damage to surrounding normal tissues. Instead, locally high temperature from microscopic level was required to achieve considerable efficiency in killing CAFs and degrading ECM.

To sum up, soft ICG@2%NGs showed more advantages in tumor penetration and distribution, due to excellent deformability to overcome obstacles from aberrant TMME. Deeper tumor penetration and more uniform distribution of soft ICG@2%NGs would benefit to higher efficiency in regulating tumor mechanics, whereas stiff ICG@15%NGs distributed nearby blood vessels. Hence, soft ICG@2%NGs would be a suitable choice for TMME modulation.

### 2.4 Softness‐Mediated Mild Hyperthermia is Potent in Regulating Tumor Mechanics by Eliminating CAFs and Degrading ECM

Despite soft ICG@2%NGs has been proved to achieve outstanding distribution in tumor tissue relying on outstanding deformability, whether the advantages of distribution did contribute to CAFs inhibition and ECM degradation should be further investigated.

**Figure 5:**
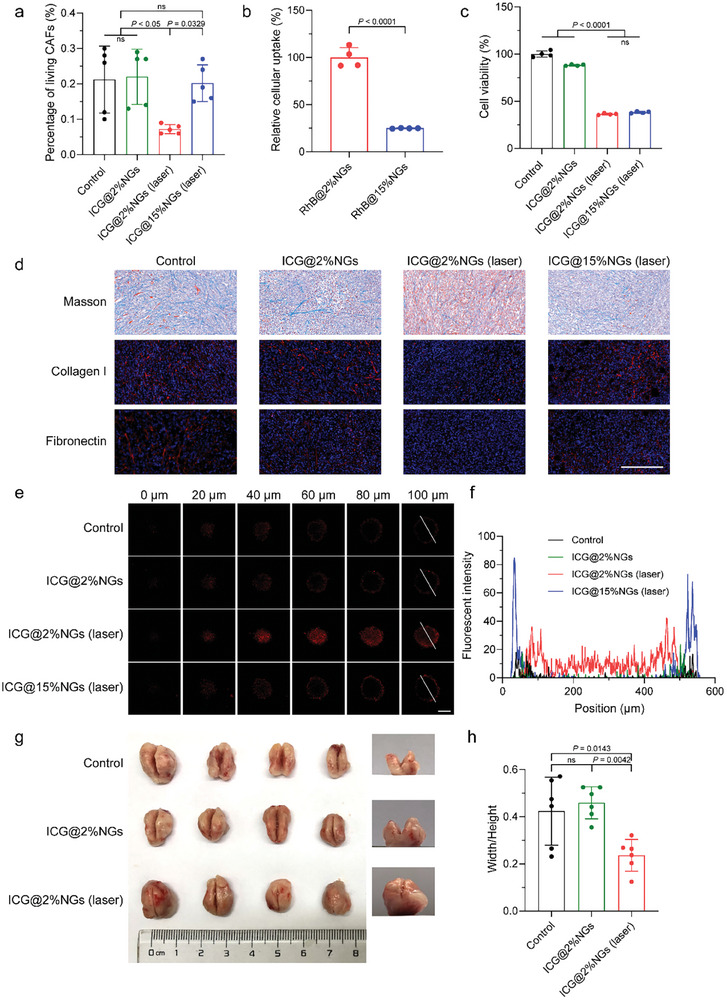
Softness‐mediated mild hyperthermia is potent in regulating tumor mechanics by eliminating CAF and degrading ECM. a) Percentage of living CAFs after mild‐PTT by ICG‐loaded nanogels with different stiffness. Data are presented as mean values ± SD (*n* = 5 biological independent replicates). b) Relative cellular uptake of RhB‐labeled nanogels with different stiffness in CAFs. Data are presented as mean values ± SD (*n* = 4 biological independent replicates). c) Viability of CAFs after mild‐PTT by ICG‐loaded nanogels with different stiffness in vitro. Data are presented as mean values ± SD (*n* = 4 biological independent replicates). d) Masson, collagen I and fibronectin staining of tumors after mild‐PTT by ICG‐loaded nanogels with different stiffness. Blue: nuclei of tumor cells. Red: collagen I or fibronectin. Scale bar = 200 µm. e) Penetration of RhB@15%NGs after mild‐PTT by ICG‐loaded nanogels with different stiffness in 3D tumor spheroids. Scale bar = 200 µm. f) Distribution of fluorescent intensity along radial direction in e). g) Solid stress of tumors after mild‐PTT by ICG@2%NGs. h) Semi‐quantification of tumor solid stress in g). Data are presented as mean values ± SD (*n* = 6 biological independent replicates). Statistical significance of a,c, h) was calculated by one‐way ANOVA. Statistical significance of b) was calculated by unpaired two‐sided Student's t‐test.

Herein, both ICG@2%NGs and ICG@15%NGs were i.v. injected and irradiated by 808 nm laser with mild temperature below 43 °C for 10 min. 24 h later, the tumors were harvested for cytometry analysis to evaluate the percentage of surviving CAFs. The gating strategy was demonstrated in Figure S15 (Supporting Information) and the results revealed that individual administration of ICG@2%NGs without laser irradiation could hardly threaten the viability of CAFs in tumor tissues (**Figure** **5**a). Nevertheless, introduction of 808 nm laser irradiation to ICG@2%NGs markedly decreased the percentage of living CAFs from (0.30 ± 0.10) % to (0.10 ± 0.03) %, as a result of outstanding permeability and uniform distribution. As for ICG@15%NGs, in spite of being irradiated by laser, negligible influence on viability of CAFs could be observed. CAF‐related markers, including α‐smooth muscle actin (α‐SMA) and fibroblast activation protein (FAP), were significantly decreased by ICG@2%NGs with laser irradiation (Figure S16, Supporting Information). Much fewer CAFs‐associated signals could be detected in the group of ICG@2%NGs with laser compared to the group of ICG@15%NGs with laser, suggesting soft ICG@2%NGs‐mediated mild‐PTT had higher efficiency in eliminating CAFs.

In our previous work, soft 2%NGs exhibited more efficient internalization by 4T1 cells and RAW 264.7 cells.^[^ ^18^ ^]^ Herein, we found that according to the ratio of 1:2.05 between fluorescent intensity of RhB@2%NGs and RhB@15%NGs as we previously reported,^[^ ^18^ ^]^ CAFs preferred soft RhB@2%NGs to stiff RhB@15%NGs, contributing to higher accumulation in tumor tissues and tumor cells (Figure 5b; Figure S17, Supporting Information). After excluding the cytotoxicity of blank nanogels (Figure S18, Supporting Information), MTT assay results showed that incubation with ICG@2%NGs led to feeble cytotoxicity (Figure 5c). After irradiation for 10 min with manually‐controlled temperature below 43 °C, ≈60% of CAFs were killed by both ICG@2%NGs and ICG@15%NGs. However, no significant difference in cell viability was observed because the vast majority of thermal was produced by ICG in culture medium and discrepancy of cellular uptake could hardly make a difference. Masson, collagen I and fibronectin staining of tumor tissues demonstrated that ICG@2%NGs without laser irradiation could hardly degrade ECM, while the density of ECM was decreased to a great extent after laser irradiation (Figure 5d; Figure S19, Supporting Information). As for ICG@15%NGs, insignificant effect of ECM degradation could be detected, as a result of poor permeability and inadequate distribution.

Presumably, nanomedicine penetration should be promoted by CAFs suppression and ECM degradation. To this end, 3D tumor spheroids were utilized to investigate whether soft ICG@2%NGs with laser irradiation was beneficial to tumor penetration of stiff RhB@15%NGs with poor permeability. Consistently, ICG@2%NGs without laser was useless whereas ICG@2%NGs with laser significantly boosted the penetrating depth of RhB@15%NGs (Figure 5e,f). Interestingly, the penetration increment of RhB@15%NGs was negligible after treatment by ICG@15%NGs with laser, but higher fluorescent intensity could be observed at tumor spheroid periphery. We also noticed that the distribution of soft RhB@2%NGs from periphery to core area of 3D tumor spheroids was barely affected by ICG@2%NGs with laser irradiation (Figure S20, Supporting Information). Consistently, the fluorescent intensity of spheroid peripheral was enhanced. Together with the result that stiff ICG@15%NGs‐mediated mild‐PTT could not promote the penetration but enhance the enrichment of stiff RhB@15%NGs in Figure 5e, we reasoned that feeble enhancement of nanomedicine penetration after treatment by photothermal agents with close stiffness, mainly resulted from similar permeability and limited progress in regulating tumor mechanics in deeper tumor tissue. Furthermore, 3D tumor spheroids without 3T3 cells, of which the matrix was much less than the above‐mentioned 3D tumor spheroids with 3T3 cells, were taken into comparison (Figure S21, Supporting Information). The results verified that RhB@15%NGs penetrated deeper in 3D tumor spheroids without 3T3 cells than that with 3T3 cells, which meant matrix was a main hindrance for deep penetration. After depletion of CAFs and degradation of ECM, variation of tumor solid stress was further investigated.^[^ ^6d,e^ ^]^ It was apparent that mild‐PTT relieved solid stress of orthotopic 4T1 tumors, whereas solid stress of tumors treated by ICG@2%NGs without laser irradiation was similar to control group (Figure 5g,h), supporting the conclusion that soft ICG@2%NGs‐mediated mild‐PTT is efficient in regulating tumor mechanics.

To further corroborate that soft ICG@2%NGs could promote mild‐PTT, the percentage of living cancer stem cells (CSCs) in tumor tissues was explored.^[^ ^6^, ^7^ ^]^ In excellent agreement with our expectation, typical CSCs markers including CD133^+^ and CD44^+^CD24^−^ proved that mild‐PTT by soft ICG@2%NGs efficiently restrained CSCs viability, whereas stiff ICG@15%NGs with laser irradiation exhibited insignificant effect (Figures S22 and S23, Supporting Information). Fluorescent images of CD133 and SOX2 staining provided the same conclusion that ICG@2%NGs was more efficient in eliminating CSCs (Figure S24, Supporting Information). Higher cellular internalization of soft 2%NGs than stiff 15%NGs was also a key factor on better photothermal efficiency (Figure S25, Supporting Information).

In summary, mild‐PTT played an important role in release of solid stress by killing CAFs and degrading ECM and promoting nanomedicine tumor penetration. For photothermal agents, soft ones (e.g., ICG@2%NGs) were more efficient, relying on better permeability, than stiff counterparts (e.g., ICG@15%NGs) in regulating abnormal TMME. Photothermal agents could hardly promote penetration of nanoparticles with similar stiffness because only matrix around the depth they both could reach would be degraded, instead of deeper matrix, and enrichment of nanoparticles would be improved rather than penetration (Figure S26a, Supporting Information). For the purpose of augmenting nanoparticles tumor penetration, photothermal agents softer than subsequent nanoparticles would be needed to permeate further and regulate TMME at deeper tumor site (Figure S26b, Supporting Information).

### 2.5 Stiff Nanomedicine Gains More Benefits in Tumor Accumulation and Antitumor Effect from Tumor Mechanics Regulation Relative to Soft Counterparts

Many studies have documented that tumor accumulation and deep penetration of nanoparticles, especially stiff nanoparticles with poor deformability, were severely hindered by abnormal TMME. In contrast, the soft counterparts could overcome the obstacles from TMME to a certain extent, relying on excellent deformability.^[^ ^17^, ^18^, ^29^ ^]^ So, it was necessary to evaluate whether both soft and stiff nanoparticles could benefit from TMME regulation.

**Figure 6:**
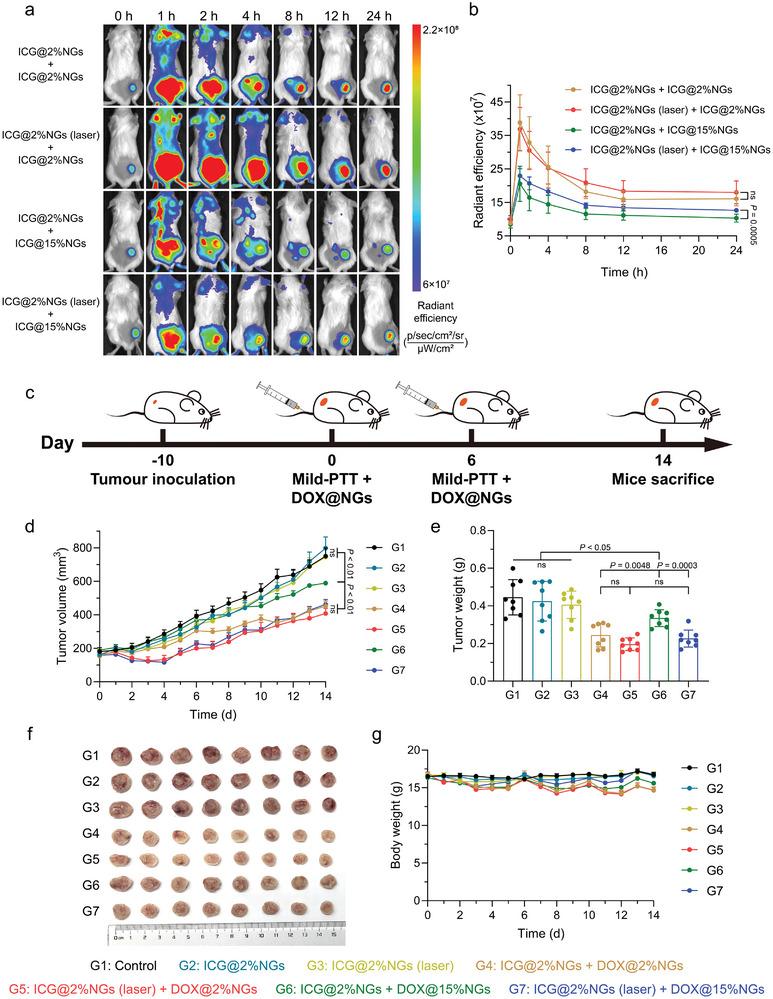
Stiff nanomedicine gains more benefits in tumor accumulation and antitumor effect from tumor mechanics regulation relative to soft counterparts. a) In vivo fluorescent images of tumor accumulation of ICG‐loaded nanogels with different stiffness after mid‐PTT by ICG@2%NGs. b) Semi‐quantification of tumor accumulation of ICG‐loaded nanogels with different stiffness in a). Data are presented as mean values ± SD (*n* = 7 biological independent replicates). c) Illustration of drug administration strategy. d) Tumor growth profiles of 4T1 tumor‐bearing mice after treatment by DOX‐loaded nanogels with different stiffness as promoted by ICG@2%NGs‐mediated mid‐PTT. Data are presented as mean values ± SEM (*n* = 8 biological independent replicates). e) Weight of tumors after treatment. Data are presented as mean values ± SD (*n* = 8 biological independent replicates). f) Photograph of tumors harvested from the mice after treatment. g) Body weight profiles of mice after treatment. Data are presented as mean values ± SEM (*n* = 8 biological independent replicates). Statistical significance was calculated by unpaired two‐sided Student's t‐test. G1, Control; G2, ICG@2%NGs; G3, ICG@2%NGs (laser); G4, ICG@2%NGs + DOX@2%NGs; G5, ICG@2%NGs (laser) + DOX@2%NGs; G6, ICG@2%NGs + DOX@15%NGs; G7, ICG@2%NGs (laser) + DOX@15%NGs.

First, we utilized 4T1 subcutaneous tumors as tumor models, ICG@2%NGs as photothermal agent, ICG‐loaded nanogels with different stiffness as model nanoparticles, to investigated how TMME modulation affected nanoparticles tumor accumulation. After 24 h post injection of ICG@2%NGs with or without mild‐PTT, soft ICG@2%NGs and stiff ICG@15%NGs were i.v. injected, respectively. The in vivo fluorescent imaging demonstrated that soft ICG@2%NGs possessed higher tumor accumulation, relative to stiff ICG@15%NGs (**Figure** **6**a,b). However, after mild‐PTT, tumor accumulation of stiff ICG@15%NGs acquired significant promotion, with increment of ≈22.6%, whereas only 11.9% enhancement was noted for ICG@2%NGs. Even so, it was noteworthy that although stiff ICG@15%NGs obtained higher promotion of tumor accumulation, the amount of soft ICG@2%NGs in tumor was still higher because of originally excellent tumor permeability.

Finally, DOX@2%NGs and DOX@15%NGs were introduced to represent soft and stiff nanomedicine to evaluate whether mild‐PTT could contribute to antitumor efficacy of nanomedicine with varied stiffness. Accordingly, 4T1 subcutaneous tumors were established and randomly divided into seven groups including control (G1), ICG@2%NGs (G2), ICG@2%NGs (laser) (G3), ICG@2%NGs + DOX@2%NGs (G4), ICG@2%NGs (laser) + DOX@2%NGs (G5), ICG@2%NGs + DOX@15%NGs (G6), ICG@2%NGs (laser) + DOX@15%NGs (G7). Among these groups, ICG@2%NGs as photothermal agent was i.v. injected and followed by 808 nm laser irradiation with manually‐controlled temperature below 43 °C for 10 min, and 24 h later, DOX@2%NGs or DOX@15%NGs were i.v. injected respectively as therapeutic agents. This operation was proceeded on day 0 and day 6 (Figure 6c). Consistent with results in Figure S9b,c (Supporting Information), solely administration of ICG@2%NGs (G2) and ICG@2%NGs with laser (G3) exhibited no observable inhibition on tumor growth (Figure 6d). In addition, ICG@2%NGs with DOX@2%NGs (G4) led to better antitumor efficacy because of higher tumor accumulation, than ICG@2%NGs with DOX@15%NGs (G6). As expected, soft DOX@2%NGs and stiff DOX@15%NGs benefited from mild‐PTT to different extents. The remarkable result was that the more enhanced decrement of ≈21.4% in tumor volume was observed in DOX@15%NGs with ICG@2%NGs‐mediated mild‐PTT (G7) than DOX@15%NGs without mild‐PTT (G6), with tumor volume even close to DOX@2%NGs post mild‐PTT (G5). In contrast, only decrement of ≈9.7% in tumor volume of DOX@2%NGs after mild‐PTT was achieved (G5 versus G4). Even though, DOX@2%NGs with mild‐PTT (G5) was still the most efficient strategy for tumor inhibition, based on superior deformability of soft 2%NGs. Similar conclusion could be obtained in tumor weight and tumor photo, that antitumor efficacy of DOX@15%NGs received a greater extent of enhancement from ICG@2%NGs‐mediated mild‐PTT, compared to soft DOX@2%NGs (Figure 6e,f). Afterwards, volume‐based as well as weight‐based tumor inhibition rates were calculated (Figure S27, Supporting Information). The harvested tumors were fixed with 4% paraformaldehyde for H&E, Caspase‐3 and Ki67 staining to evaluate necrosis, apoptosis and proliferation of tumor cells. Regularly, sparer distribution of tumor cells, higher percentage of apoptotic cells and lower percentage of proliferative cells could be observed in groups with better antitumor efficacy, and G5 was still the most efficient group (Figure S28, Supporting Information). Consistently, body weight proved that mild‐PTT was a safe adjuvant treatment (Figure 6g). Therapeutic agents slightly affected body weight of mice which recovered to normal level after treatment and no abnormal tissue was observed in H&E staining (Figure S29, Supporting Information). All the physiological indicators of blood biochemical analysis and blood routine examine were in the normal range, among which the number of WBC decreased with increased antitumor efficacy (Figure S30, Supporting Information).

In conclusion, ECM degradation did promote nanoparticles tumor penetration and stiff counterparts possessed poor permeability could benefit more from TMME regulation. According to 3D tumor spheroid models (Figure 5e; Figure S20, Supporting Information), the reason for restrained tumor accumulation increment of 2%NGs after ICG@2%NGs‐mediated mild‐PTT might be the similar stiffness. Because of close permeability, ICG@2%NGs could only degrade the ECM at the depth which 2%NGs could penetrate to and enrichment of 2%NGs in such area was increased. No further degradation of ECM at deeper regions led to limited improvement in penetration of 2%NGs. On the other hand, evident boost of 15%NGs tumor accumulation relied on both higher enrichment and deeper penetration. It needed to be mentioned that, after mild‐PTT tumor accumulation of stiff 15%NGs was still lower than soft 2%NGs, but the antitumor efficiency demonstrated no significant difference. The reason might be that, for G4 and G5, soft ICG@2%NGs could only enhance the enrichment of soft DOX@2%NGs at original depth, where DOX@2%NGs could penetrate in the absence of mild‐PTT. As a result, the concentration of DOX@2%NGs at such depth increased and exceeded cytotoxic dosage for elimination of tumor cells. Meanwhile, limited elimination of tumor cells in deeper tumor region resulted in restricted improvement in antitumor effect of G5, as compared to G4 (Figure S31a, Supporting Information). However, in G6 and G7, the penetration of stiff DOX@15%NGs was markedly improved post soft ICG@2%NGs‐mediated mild‐PTT and tumor cells further away from blood vessels could be killed, leading to more pronounced antitumor efficacy of G7, which was almost equivalent to G4 and G5 (Figure S31b, Supporting Information).

## 3 Conclusion

In summary, we prepared two kinds of nanogels with distinctive stiffness, the soft 2%NGs and the stiff 15%NGs, and utilized these nanogels to load ICG as photothermal agents for mild‐PTT. Owe to excellent deformability and permeability, ICG@2%NGs could distribute more uniformly along blood vessels and penetrate more deeply in tumor tissue. As a result, soft ICG@2%NGs exhibited higher efficiency in depleting CAFs and degrading ECM than stiff ICG@15%NGs. Even so, penetration of 2%NGs was not enhanced by ICG@2%NGs‐mediated mild‐PTT, and consistent conclusion could be drawn from 15%NGs and ICG@15%NGs. However, nanogels enrichment at original area where they could arrive without the introduction of mild‐PTT, was significantly increased after mild‐PTT by photothermal agents with close stiffness, because only matrix at the same depth was degraded. In sharp contrast, soft ICG@2%NGs conspicuously boosted the penetration and enrichment of 15%NGs. So, we could conclude that photothermal agents with lower stiffness should be applied first to penetrate deeper tumor site and degrade deeper matrix, removing handicap of the aberrant TMME and paving the paths for subsequent stiff nanomedicines to internal tumor cells. This study reveals the crucial role of nanomedicine mechanical properties in drug delivery and provides a novel strategy for overcoming barriers in solid tumors with soft nanogels, having significant implications for therapies of solid malignancies and treatments of fibrotic diseases.

## Conflict of Interest

The authors declare no conflict of interest.

## Author Contributions

Z.L. and Y.Z. contributed equally to this work. Z.F.L. designed and supervised the project. Z.F.L. and X.L.Y. acquired financial support. Z.L., Y.B.Z., Z.J.Z., H.M.W, C.W., C.X., S.Y.L and S.Y.Z performed experiments. Z.L., Y.B.Z. and Z.F.L. analyzed and interpreted the data. Z.L. and Z.F.L. wrote and revised the manuscript.

## Supporting information

Supporting Information

## Data Availability

The data that support the findings of this study are available from the corresponding author upon reasonable request.
