# Supplementary material for: Softness‐Aided Mild Hyperthermia Boosts Stiff Nanomedicine by Regulating Tumor Mechanics
Source: Adv Sci (Weinh). 2024 May 5;11(26):2306730. doi: 10.1002/advs.202306730 (PMC11234402; doi:10.1002/advs.202306730)
Supplement: Supplementary file 1 — Supporting Information [file ADVS-11-2306730-s001.pdf]

ADVANCED SCIENCE
Open Access

Supporting Information

for *Adv. Sci.*, DOI 10.1002/advs.202306730

Softness-Aided Mild Hyperthermia Boosts Stiff Nanomedicine by Regulating Tumor Mechanics

*Zheng Li, Yabo Zhu, Zhijie Zhang, Huimin Wang, Chong Wang, Chen Xu, Shiyou Li, Shuya Zhang, Xiangliang Yang and Zifu Li**

Supporting Information

Softness-aided mild hyperthermia boosts stiff nanomedicine by regulating tumor mechanics

Zheng Li, Yabo Zhu, Zhijie Zhang, Huimin Wang, Chong Wang, Chen Xu, Shiyou Li, Shuya Zhang, Xiangliang Yang, and Zifu Li*

Z. Li, Y. Zhu, Z. Zhang, H. Wang, Dr. C. Wang, Dr. C. Xu, S. Li, S. Zhang

Department of Nanomedicine and Biopharmaceuticals

College of Life Science and Technology

Huazhong University of Science and Technology

Wuhan, 430074, P. R. China

Professor X.L. Yang and Professor Z.F. Li

[1] Department of Nanomedicine and Biopharmaceuticals, College of Life Science and Technology, Huazhong University of Science and Technology, Wuhan, 430074, P. R. China

[2] National Engineering Research Center for Nanomedicine, Huazhong University of Science and Technology, Wuhan, 430074, P. R. China

[3] Key Laboratory of Molecular Biophysics of Ministry of Education, Huazhong University of Science and Technology, Wuhan, 430074, P. R. China

[4] Hubei Key Laboratory of Bioinorganic Chemistry and Materia Medical, Huazhong University of Science and Technology, Wuhan, 430074, P. R. China

[5] Hubei Engineering Research Center for Biomaterials and Medical Protective Materials, Huazhong University of Science and Technology, Wuhan, 430074, P. R. China

[6] Hubei Bioinformatics and Molecular Imaging Key Laboratory, Huazhong University of Science and Technology, Wuhan, 430074, P. R. China

E-mail: zifuli@hust.edu.cn

1. Experimental Section

Materials. N-isopropylmethacrylamide (NIPMAM) and N,N'-bis(acryloyl)cystamine (BAC)

were purchased from Sigma-Aldrich LLC. (St Louis, MO, USA). Methacrylic acid (MAA) was purchased from Sinopharm Chemical Reagent Co., Ltd. (Shanghai, China). Potassium persulfate (KPS) and sodium dodecyl sulfate (SDS) were purchased from Aladdin Reagent Inc. (Shanghai, China). Doxorubicin·HCl (DOX·HCl) was purchased from Meilun Biotech Co., Ltd. (Dalian, China). Indocyanine Green (ICG) was purchased from J&K Scientific Ltd. (Beijing, China). Rhodamine B (RhB), 4-dimethylaminopyridine (DMAP), N,N'-Dicyclohexylcarbodiimide (DCC) and 2-hydroxyethyl methacrylate (HEMA) were purchased from Energy Chemical (Shanghai, China). Flow cytometry antibodies including FITC anti-mouse CD45 (103107), PE anti-mouse CD326 (EpCAM) (118206), PE/Cyanine7 anti-mouse CD31 (102417), APC anti-mouse TER-119/Erythroid cells (116212), Brilliant Violet 421™ anti-mouse Podoplanin (127423), APC anti-mouse CD133 (141208), FITC anti-mouse CD24 (101806), PE/Cyanine7 anti-mouse/human CD44 (103030) and Zombie Red™ Fixable Viability Kit (423109) were purchased from Biolegend Inc. (San Diego, CA. USA).

Cell culture and animals. The murine breast cancer cell line 4T1 was acquired from Shanghai Institutes for Biological Sciences. The NIH-3T3 cell line was acquired from National Collection of Authenticated Cell Cultures. The 4T1 cells and NIH-3T3 cells were cultured in RPMI 1640 medium supplemented with 10% fetal bovine serum and 1% antibiotics (penicillin: 100 U/mL, streptomycin: 100 μg/mL) at 37 °C under a 5% CO_2 atmosphere.

BALB/c mice (female) were purchased from Vital River Laboratory Animal Technology Co. Ltd. (Beijing, China). Mice were housed in an animal facility under constant environmental conditions (room temperature, 21 ± 1 °C; relative humidity, 40−70%, and a 12-h light-dark cycle). All mice had access to food and water ad libitum. All animal experiments were approved by the Institutional Animal Care and Use Committee at Tongji Medical College, Huazhong University of Science and Technology (Wuhan, China). The experiment protocols were approved by the Institutional Animal Ethical Committee of the Huazhong University of Science and Technology (Wuhan, China). The animal ethical clearance project number is 2019S924.

Synthesis of P(NIPMAM-ss-MAA) nanogels with different stiffness. Emulsion

polymerization was used to synthesize P(NIPMAM-ss-MAA) nanogels with different stiffness. To obtain soft 2%NGs, 550 mg of NIPMAM, 18.65 μL of MAA, 35 mg of SDS and 22.5 mg of BAC pre-dissolved by ethyl alcohol were added to 80 mL of water in a three-necked round-bottom flask. As for stiff 15%NGs, 20 mg of SDS and 168.9 mg of BAC were added, instead. The solution was vacuumized for 10 min and then inflated with argon for three turns to remove oxygen and ethyl alcohol. The solution was heated to 80 °C, then 10 mg of KPS was dissolved and injected to the solution to initiate the polymerization. After polymerization for 6 h with stirring, the solution was cooled to room temperature. Unpolymerized monomers and small molecules were removed via ultrafiltration (molecular weight cutoff 10,000) and the nanogels were washed by water for three times. Finally, purified nanogel solution was concentrated to 20 mg/mL for storage at 4 °C and further applications.

So far, soft 2%NGs and stiff 15%NGs were obtained.

Preparation of ICG-loaded nanogels. 5 mL aqueous solution with DOX·HCl concentration of 2 mg/mL was added to 5 mL of nanogels solution and stirred for 48 h. Free DOX was removed via ultrafiltration (molecular weight cutoff 10,000) and DOX-loaded nanogels were concentrated to 1 mg/mL of DOX. The concentration of DOX was detected via UV–vis spectroscopy at 483 nm.

5 mL aqueous solution with ICG concentration of 1 mg/mL was added to 5 mL of DOX-loaded nanogels solution and stirred for 48 h. Free ICG was removed via ultrafiltration (molecular weight cutoff 10,000) and ICG-loaded nanogels were concentrated to 1 mg/mL of ICG. The concentration of ICG was detected via UV–vis spectroscopy at 783 nm.

So far, soft ICG@2%NGs and stiff ICG@15%NGs were obtained.

Synthesis of Rhodamine-labeled nanogels. 5 g of Rhodamine B (10.45 mmol), 75 mg of DMAP (0.615 mmol), and 2.6 g of DCC (12.6 mmol) were dissolved in 52.5 mL of anhydrous CH_2Cl_2. The solution was vacuumed and inflated with argon to remove oxygen. After stirring for 30 min under argon protection, 1.55 mL HEMA (12.5 mmol) was added. After reaction for 25 h at 20 °C under argon protection, the product was purified by column chromatography on silica gel with 90/10 DCM/MeOH eluent and concentrated on rotary evaporator to remove solvent and obtain dry RhB-HEMA powder.

Similar to the synthesis of P(NIPMAM-ss-MAA), 255 μg of RhB-HEMA was added to water together with other components, followed by the same procedure. Purified Rhodamine-labeled nanogels solution was concentrated to 20 mg/mL for storage at 4 °C and further application.

So far, soft RhB@2%NGs and stiff RhB@15%NGs were obtained.

Preparation of DOX-loaded nanogels. 5 mL aqueous solution with DOX·HCl concentration of 2 mg/mL was added to 5 mL of nanogels solution and stirred for 48 h. Free DOX was removed via ultrafiltration (molecular weight cutoff 10,000) and DOX-loaded nanogels were concentrated to 1 mg/mL of DOX. The concentration of DOX was detected via UV–vis spectroscopy at 483 nm, and the drug loading capacity was around 5%.

So far, soft DOX@2%NGs and stiff DOX@15%NGs were obtained.

Characterization of nanogels and ICG-loaded nanogels. The hydrodynamic diameter distribution was measured in PBS buffer at 37 °C by dynamic light scattering (DLS, Malvern, Zetasizer Nano-ZS, UK), and the zeta potential was measured in water at 37 °C. The data was collected with Zetasizer Software (Ver. 7.13).

TEM images were captured by transmission electron microscopy (TEM, JEOL, JEM-1230, Japan). 10 μL of nanogel solution with concentration of 0.01 mg/mL was dropped onto a 400-mesh carbon-coated copper grid and dried for 24 h. Then the nanogels were stained with phosphomolybdic acid solution with concentration of 10 mg/mL for 2 min and washed by water for 1 min. Then the nanogels were imaged by TEM after drying.

AFM images were captured by atomic force microscopy (AFM, Bruker, Multimode 8, Germany) in air phase. 50 μL of nanogel solution with concentration of 0.01 mg/mL was dropped onto silicon slice. After drying for 24 h, the nanogels were imaged by AFM with contact mode.

Young's modulus was detected by AFM in liquid phase. The silicon slice was soaked in polyethyleneimine (PEI) solutions for modification with positive charge. 50 μL of nanogel solution with concentration of 0.01 mg/mL was dropped onto silicon slice and maintained for 10 min to absorb onto the slice. The force curve was captured in liquid phase by AFM with tapping mode and Young's modulus was calculated with NanoScope Analysis (Ver. 1.5) software.

Temperature-responsiveness and pH-responsiveness were measured by DLS. Range of temperature-responsiveness was from 25 to 55 °C and interval was 1 °C with equilibration time of 60 s. Range of pH-responsiveness was from 3 to 9 and interval was 1 with equilibration time of 600 s. GSH-responsiveness was detected by TEM. Nanogels were incubated with 10 mmol/L GSH solution for 24 h and morphology variation was observed by TEM.

Photostability of ICG@2%NGs. UV-vis absorption of ICG dissolved in H_2O, ICG@2%NGs dispersed in H_2O and ICG dissolved in DMSO were detected by UV-vis absorption spectra with ICG concentration of 20 μg/mL. The range of wavelength was from 500 nm to 900 nm.

Photothermal properties of ICG@2%NGs and free ICG in H_2O with ICG concentration of 20 μg/mL was characterized via irradiation by 808 nm laser with power of 1 W/cm^2 for 8 min. The laser area was 0.75 cm^2 and the distance over samples was 2 cm. The temperature was detected and the infrared image was captured by the infrared camera with time interval of 1 min.

Photostability of ICG@2%NGs and free ICG in H_2O with ICG concentration of 20 μg/mL was characterized via temperature increment with multiple-round irradiation and UV-vis absorption after irradiation. The samples were irradiated by 808 nm laser with power of 1 W/cm^2 for 8 min and cooled down to room temperature. This operation was circulated for three rounds and the temperature was detected with time interval of 1 min. After irradiation by 808 nm laser with power of 1 W/cm^2 for 30 s, 60 s, 90 s and 180 s, UV-vis absorption of samples was detected by UV-vis absorption spectra.

In vitro photothermal properties of ICG@2%NGs. Concentration-dependent photothermal properties of ICG@2%NGs in H_2O with ICG concentration of 0 μg/mL, 10 μg/mL, 20 μg/mL and 40 μg/mL were characterized via irradiation by 808 nm laser with power of 1 W/cm^2 for 8 min. The temperature was detected and the infrared image was captured by the infrared camera with time interval of 1 min.

Power-dependent photothermal properties of ICG@2%NGs in H_2O with ICG concentration of 20 μg/mL were characterized via irradiation by 808 nm laser with power of 0.5 W/cm^2, 1 W/cm^2 and 1.5 W/cm^2 for 8 min. The temperature was detected and the infrared image

was captured by the infrared camera with time interval of 1 min.

Blood perfusion after PTT. 4T1 orthotopic tumor models were established via injection of 5 × 10^5 4T1 cells in 50 μL of PBS. The mice were i.v. injected ICG@2%NGs with ICG dosage of 4 mg/kg, when the tumor volume reached around 500 mm^3. 4 h later, the mice were randomly divided into mild-PTT group and hyper-PTT group. The mice were anesthetic and the tumors were imaged by laser speckle blood flow imager (Perimed: PeriCam PSI System) to detect blood perfusion before 808 nm laser irradiation. Then, the tumors were irradiated by 808 nm laser. The laser area was 0.75 cm^2 and the distance over tumors was 2 cm. We started the laser irradiation with power of 1 W/cm^2 and monitored tumor temperature with infrared thermal imager. For mild-PTT group, the tumor temperature could reach 43 °C within 30 s. When the tumor temperature reached 43 °C, the laser power was turned down and adjusted manually to maintain the temperature below 43 °C. In detail, when the tumor temperature started to decrease, the laser power was increased; on the contrary, the laser power was decreased when the tumor temperature reached 43 °C. As for hyper-PTT group, the tumors were continuously irradiated by laser with power of 1 W/cm^2 and the tumor temperature would increase over 50 °C. The whole irradiation process retained for 10 min. After removal of laser, the tumors were imaged immediately. The images of blood perfusion before and after irradiation were semi-quantified by PIMSoft (Ver. 2.0.3) software and the variation was evaluated.

Effects of blood perfusion on tumor accumulation. 4T1 subcutaneous tumor models were established via injection of 1 × 10^6 4T1 cells in 100 μL of PBS. The mice were i.v. injected ICG@2%NGs with ICG dosage of 4 mg/kg, when the tumor volume reached around 200 mm^3. 4 h later, the mice were randomly divided into control group, mild-PTT group and hyper-PTT group and i.v. injected RhB@15%NGs with dosage of 100 mg/kg. Then, the tumors were irradiated by 808 nm laser for 10 min with manually-controlled temperature below 43 °C in mild-PTT group by regulating power of laser or over 50 °C in hyper-PTT group by controlling power of laser at 1 W/cm^2. Control group was not irradiated. After removal of laser, the mice were sacrificed and the tumors were harvested for ex vivo imaging by in vivo imaging system (PerkinElmer, IVIS Lumina XR, USA). The fluorescent intensity was quantified with Living Image (Ver. 4.0) software. The harvested tumors were

fixed with 4% paraformaldehyde and stained with fluorescence-labeled anti-CD31 antibody (1:50 for dilution) to evaluate penetration and length of blood vessels after mild-PTT or hyper-PTT. The penetration distance was evaluated by measuring the distance from RhB fluorescent signal to the nearest CD31 fluorescent signal. The fluorescent images were analyzed with CaseViewer (Ver. 2.4) and ImageJ (Ver. 1.52) software.

Effects of blood perfusion on nanomedicine antitumor efficacy. 4T1 subcutaneous tumor models were established via injection of 1 × 10^6 4T1 cells in 100 μL of PBS. The mice were randomly divided into control group (G1), ICG@2%NGs group (G2), ICG@2%NGs (laser) group (G3), ICG@2%NGs + Doxil group (G4), ICG@2%NGs (laser) + Doxil group (G5) when the tumor volume reached around 150 mm^3. G2-G5 were i.v. injected ICG@2%NGs with ICG dosage of 4 mg/kg. 4 h later, G4 and G5 were i.v. injected Doxil with DOX dosage of 3 mg/kg. Then, G2-G5 were irradiated by 808 nm laser for 10 min with manually-controlled temperature below 43 °C. This operation was proceeded on day 1 and day 7. The length and width of tumors were measured every day for 15 days and the tumor volume was calculated according to the formula: tumor volume = length × $width^2/2$. Meanwhile, the body weight was recorded. When the experiment was finished, the mice were sacrificed and tumors were harvested and weighed. Then the photo of tumors was captured. Afterwards, the tumors were fixed with 4% paraformaldehyde for H&E, Caspase-3 and Ki67 staining and the scanning images were analyzed with CaseViewer (Ver. 2.4) and ImageJ (Ver. 1.52) software to evaluate the percentage of necrosis, apoptosis and proliferation of tumor cells. The major organs including heart, liver, spleen, lung and kidney and the blood of mice were collected to evaluate the safety of therapeutics. The major organs were fixed to evaluate the histological toxicity and the blood was for blood biochemical analysis as well as blood routine examine to evaluate the treatment toxicity.

In vitro stiffness-dependent penetration. In vitro stiffness-dependent penetration was evaluated with 3D tumor spheroids. RPMI medium with 10% of fetal bovine serum (FBS), 1% of antibiotics (100 U/mL of penicillin and 100 μg/mL of streptomycin) and 1.2% of methylcellulose was prepared. NIH-3T3 cells and 4T1 cells were mixed in the ratio of 1:2 with concentration of 1 × 10^6/mL, and 25% volume of above medium with methylcellulose

was added. 25 µL of mixed cells were seeded on the internal surface of culture dish lid. Then the lid was flipped and covered on the culture dish with PBS. Medium drops were suspended and 3D tumor spheroids formed after 36 h. Then the tumor spheroids were washed off with PBS and transferred into new medium with 100 µg/mL of RhB@2%NGs or RhB@15%NGs. After 2 h of culturing, the tumor spheroids were transferred to PBS and the distribution of fluorescent intensity was detected by confocal laser scanning microscope (Olympus, FV3000, Japan). The images were analyzed with FluoView31S (Ver. 2.3) and ImageJ (Ver. 1.52) software.

In vivo stiffness-dependent tumor accumulation. 4T1 subcutaneous tumor models were established as described above. The mice were randomly divided into ICG@2%NGs group and ICG@15%NGs group, when the tumor volume reached around 200 mm^3. The mice were i.v. injected ICG@2%NGs or ICG@15%NGs with ICG dosage of 4 mg/kg. Then the mice were imaged by in vivo imaging system (PerkinElmer, IVIS Lumina XR, USA) at 1 h, 2 h, 4 h and 8 h after drug administration. The fluorescent intensity was quantified with Living Image (Ver. 4.0) software. At the end of experiment, the mice were sacrificed and the tumors were harvested. The tumors were fixed with 4% paraformaldehyde and stained with fluorescence-labeled anti-CD31 antibody (1:50 for dilution) to evaluate penetration of ICG@2%NGs and ICG@15%NGs. The penetration distance was evaluated by measuring the distance from ICG fluorescent signal to the nearest CD31 fluorescent signal. The fluorescent images were analyzed with CaseViewer (Ver. 2.4) and ImageJ (Ver. 1.52) software.

In vivo stiffness-dependent photothermal properties. 4T1 subcutaneous tumor models were established as described above. The mice were randomly divided into control group, ICG@2%NGs group and ICG@15%NGs group, when the tumor volume reached around 200 mm^3. The mice were i.v. injected ICG@2%NGs or ICG@15%NGs with ICG dosage of 4 mg/kg. 4 h later, the tumors were irradiated by 808 nm laser with power of 1 W/cm^2 for 10 min. The temperature was detected and the infrared image was captured by the infrared camera with time interval of 1 min.

In vivo depletion of CAFs and CSCs. 4T1 subcutaneous tumor models were established as described above. The mice were randomly divided into control group, ICG@2%NGs

group, ICG@2%NGs (laser) group and ICG@15%NGs (laser) group, when the tumor volume reached around 200 mm^3. The mice were i.v. injected ICG@2%NGs or ICG@15%NGs with ICG dosage of 4 mg/kg. 4 h later, the tumors were irradiated by 808 nm laser for 10 min with manually-controlled temperature below 43 °C. 24 h later, the mice were sacrificed and the tumors were harvested. The tumors were cut into fragments and incubated with collagenase IV and DNase I for 1.5 h at 37 °C under a 5% CO_2 atmosphere. Then the separated cells were infiltered through cell strainers with pore size of 70 μm to obtain single cells. The single cells were washed with PBS and divided into three groups to detect CAFs and CSCs. The first group was stained with molecular probe Zombie Red, anti-CD31, anti-CD45, anti-EpCAM, anti-TER-119 and anti-PDPN antibody. The living cells were determined by Zombie Red^- cells. Then the living CAFs were separated by exclusion of $CD31^+$ endothelial cells, $CD45^+$ lymphoid cells, $EpCAM^+$ epithelioid cells, TER-119^+ erythroid cells, and further identified by CAF-specific markers PDPN. The living cells in second and third group also determined by the portion of Zombie Red^- cells. Then the second group was stained with anti-CD133 antibody and the third group was stained with anti-CD44 and anti-CD24 antibody. The living CSCs were further identified by $CD133^+$ cells and $CD44^+$ and $CD24^-$ cells. Finally, the percentage of living CAFs and CSCs in all events were evaluated.

Consistent with above treatments, the harvested tumors were fixed with 4% paraformaldehyde. Masson staining, collagen I immunofluorescent staining and fibronectin immunofluorescent staining were used to evaluate ECM. FAP and α-SMA immunofluorescent staining were used to evaluate CAFs. CD133 and SOX2 immunofluorescent staining were used to evaluate CSCs. The scanning images were analyzed with CaseViewer (Ver. 2.4) and ImageJ (Ver. 1.52) to evaluate the percentage of ECM, CAFs and CSCs.

MTT assay in CAFs. The cytotoxicity of blank nanogels with different stiffness was evaluated by MTT assay. NIH-3T3 cells were pre-treated with TGF-β1 with concentration of 10 ng/mL for 24 h to obtain CAFs. CAFs were seeded into a 96-well plate with density of 5×10^3 cells/well for further 12 h of incubation. Serum-free medium containing blank nanogels with different concentration was added to replace original medium. After

incubation for 24 h, new serum-free medium with 1 mg/mL MTT was added to replace nanogel-contained medium for additional 2 h of incubation. Then the medium was removed and 150 μL of DMSO was added. The plate was shaken horizontally for 5 min. After the formazan crystal was dissolved, the absorption value of solutions was measured by microplate reader (Molecular Devices, Flex Station 3, USA) with wavelength of 570 nm and cell viability was further calculated.

The cytotoxicity of mild-PTT by ICG@2%NGs or ICG@15%NGs was evaluated by MTT assay. Similar to above operation, after CAFs were seeded for 12 h serum-free medium containing ICG@2%NGs or ICG@15%NGs with ICG concentration of 10 μg/mL was added for 1 h of incubation. Then, the cells were irradiated by 808 nm laser for 5 min with manually-controlled temperature below 43 °C. After irradiation, the medium with ICG@2%NGs or ICG@15%NGs was removed and new medium was added for another 24 h of incubation. Finally, MTT assay was used to evaluate viability of CAFs.

In vitro cellular uptake. CAFs were prepared as mentioned above. CAFs were seeded into a 6-well plate with density of 5 × 10^5 cells/well for further 12 h of incubation. Serum-free medium containing RhB@2%NGs or RhB@15%NGs with concentration of 100 μg/mL was added to replace original medium. After incubation for 4 h, the cells were washed by PBS for three times and collected for flow cytometry analysis (Berkman Coulter, CytoFLEX, USA). The data was analyzed with CytExpert (Ver. 2.4) and relative cellular uptake was calculated according to relative fluorescent intensity of RhB@2%NGs and RhB@15%NGs. Normally-cultured 4T1 cells were removed to 25 cm^2 ultralow-attachment cell culture flasks with serum-free DMEM/F12 culture medium containing B27, 20 ng/mL of hEGF, 20 ng/mL of bFGF, 0.4% of low endotoxin bovine serum albumin and 4 μg/mL of insulin, for 3 d of incubation to obtain CSCs. The obtained CSCs were digested and seeded to ultralow-attachment 6-well plate with density of 5 × 10^5 cells/well for further 12 h of incubation. Serum-free medium containing RhB@2%NGs or RhB@15%NGs with concentration of 100 μg/mL was added to replace original medium. After incubation for 4 h, the cells were washed by PBS for three times and collected for flow cytometry analysis and relative cellular uptake was calculated.

In vitro penetration after mild-PTT by ICG-loaded nanogels with different stiffness.

In vitro stiffness-dependent ECM degradation was evaluated with 3D tumor spheroids, which were prepared as described above. The tumor spheroids were washed off with PBS and transferred into new medium with 10 μg/mL of ICG@2%NGs or ICG@15%NGs for 2 h of incubation. The tumor spheroids in laser groups were irradiated by 808 nm laser for 10 min with manually-controlled temperature below 43 °C. Other groups were not irradiated. After irradiation, the tumor spheroids were washed by PBS and further transferred to new medium with 100 μg/mL of RhB@2%NGs or RhB@15%NGs. After 2 h of culturing, the tumor spheroids were transferred to PBS and the distribution of fluorescent intensity was detected by confocal laser scanning microscope (Olympus, FV3000, Japan). The images were analyzed with FluoView31S (Ver. 2.3) and ImageJ (Ver. 1.52) software.

Effects of ECM on penetration was evaluated using 3D tumor spheroids with or without NIH-3T3 cells. The tumor spheroids were washed off with PBS and transferred into new medium with 100 μg/mL of RhB@15%NGs. After 2 h of culturing, the tumor spheroids were transferred to PBS and the distribution of fluorescent intensity was detected by confocal laser scanning microscope and analyzed with FluoView31S (Ver. 2.3) and ImageJ (Ver. 1.52) software.

Tumor solid stress after mild-PTT by ICG@2%NGs. 4T1 orthotopic tumor models were established as described above. The mice were randomly divided into control group, ICG@2%NGs group and ICG@2%NGs (laser) group, when the tumor volume reached around 500 mm^3. The mice were i.v. injected ICG@2%NGs with ICG dosage of 4 mg/kg. 4 h later, the tumors were irradiated by 808 nm laser for 10 min with manually-controlled temperature below 43 °C. 24 h later, the mice were sacrificed and the tumors were harvested. The tumors were cut to 2/3 depth and soaked in PBS for 10 min. Then the width and depth of opening were measured in PBS and tumor solid stress was calculated according to the formula: tumor solid stress=width/depth.

In vivo tumor accumulation of nanogels with different stiffness after mild-PTT. 4T1 subcutaneous tumor models were established as described above. The mice were randomly divided into ICG@2%NGs + ICG@2%NGs group, ICG@2%NGs (laser) + ICG@2%NGs group, ICG@2%NGs + ICG@15%NGs group and ICG@2%NGs (laser) + ICG@15%NGs group, when the tumor volume reached around 200 mm^3. The mice were

i.v. injected ICG@2%NGs with ICG dosage of 4 mg/kg. 4 h later, the tumors in mild-PTT groups were irradiated by 808 nm laser for 10 min with manually-controlled temperature below 43 °C. Other groups were not irradiated. 24 h later, the mice were i.v. injected with ICG@2%NGs or ICG@15%NGs with ICG dosage of 4 mg/kg. Then the mice were imaged by in vivo imaging system (PerkinElmer, IVIS Lumina XR, USA) at 1 h, 2 h, 4 h, 8 h, 12 h and 24 h after administration. The fluorescent intensity was quantified with Living Image (Ver. 4.0) software.

Antitumor efficacy of DOX-loaded nanogels with different stiffness after mild-PTT. 4T1 subcutaneous tumor models were established as described above. The mice were randomly divided into control group (G1), ICG@2%NGs group (G2), ICG@2%NGs (laser) group (G3), ICG@2%NGs + DOX@2%NGs group (G4), ICG@2%NGs (laser) + DOX@2%NGs group (G5), ICG@2%NGs + DOX@15%NGs group (G6) and ICG@2%NGs (laser) + DOX@15%NGs group (G7) when the tumor volume reached around 150 mm^3. G2-G7 were i.v. injected ICG@2%NGs with ICG dosage of 4 mg/kg. 4 h later, G3, G5 and G7 were irradiated by 808 nm laser for 10 min with manually-controlled temperature below 43 °C. 24 h later, G4 and G5 were i.v. injected DOX@2%NGs with DOX dosage of 4 mg/kg, G6 and G7 were i.v. injected DOX@15%NGs with DOX dosage of 4 mg/kg. This operation was proceeded on day 0 and day 6. The tumor volume and the body weight were recorded every day for 15 days. At the end of the experiment, the mice were sacrificed and tumors were harvested and weighed. Then the photo of tumors was captured. The tumors were fixed with 4% paraformaldehyde for H&E, Caspase-3 and Ki67 staining and the scanning images were analyzed with FluoView31S (Ver. 2.3) and ImageJ (Ver. 1.52) software to evaluate the percentage of necrosis, apoptosis and proliferation of tumor cells. The major organs including heart, liver, spleen, lung and kidney and the blood of mice were collected to evaluate the safety of therapeutics. The major organs were fixed for H&E staining to evaluate the histological toxicity and the blood was for blood biochemical analysis as well as blood routine examine to evaluate the treatment toxicity.

Statistical Analysis

Most measured data were displayed directly and normalized data were described as relative value. Data were presented as mean values ± SD or mean values ± SEM. Sample

size for each statistical analysis was no less than 3. Statistical significance was calculated by unpaired two-sided Student's t-test between two groups and one-way ANOVA for comparison of multiple groups. P-values of <0.05 were considered statistically significant. Statistical analysis was performed using GraphPad Prism 8.0 software.

2. Supporting Figures

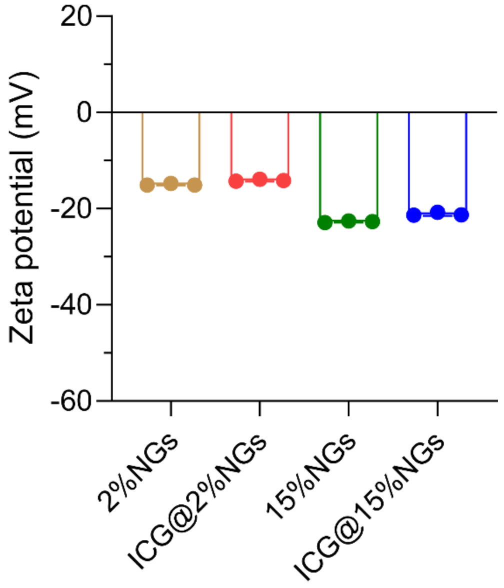

Figure S1. Zeta potential of ICG-loaded nanogels with different stiffness in H_2O. Data are presented as mean values ± SD (*n* = 3 independent replicates).

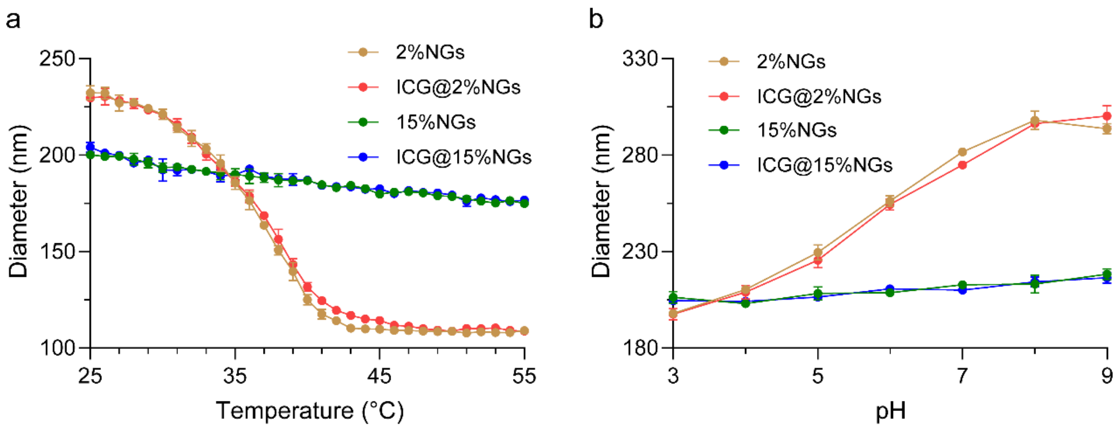

Figure S2. Stimuli-responsiveness of ICG-loaded nanogels with different stiffness in H_2O. a) Temperature-responsiveness of ICG-loaded nanogels with different stiffness in H_2O. Data are presented as mean values ± SD (*n* = 3 independent replicates). b) pH-responsiveness of ICG-loaded nanogels with different stiffness in H_2O. Data are presented

as mean values ± SD (n = 3 independent replicates).

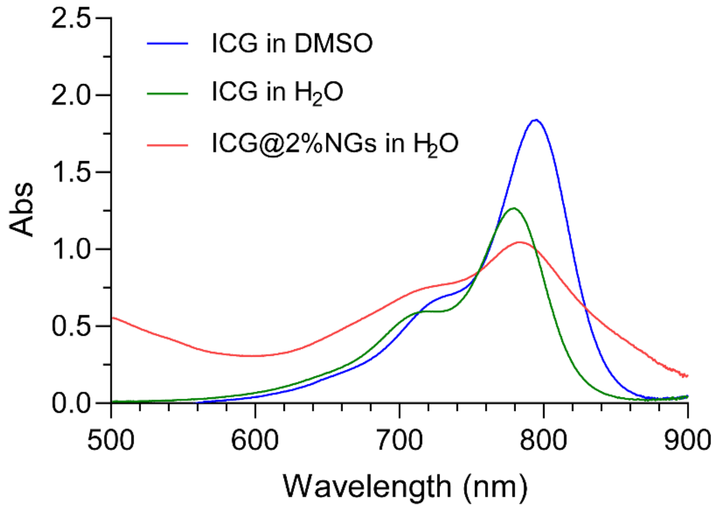

Figure S3. UV-vis absorption spectra of free ICG and ICG@2%NGs.

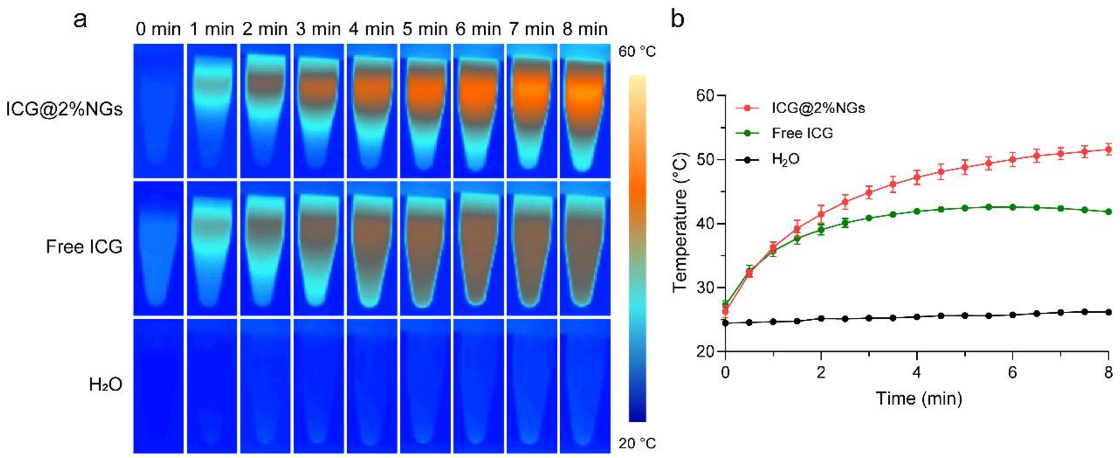

Figure S4. Photothermal properties of free ICG and ICG@2%NGs. a) Infrared images of free ICG and ICG@2%NGs in H_2O with ICG concentration of 20 μg/mL and 808 nm laser power of 1 W/cm^2. b) Photothermal properties of free ICG and ICG@2%NGs in H_2O. Data are presented as mean values ± SD (n = 3 independent replicates).

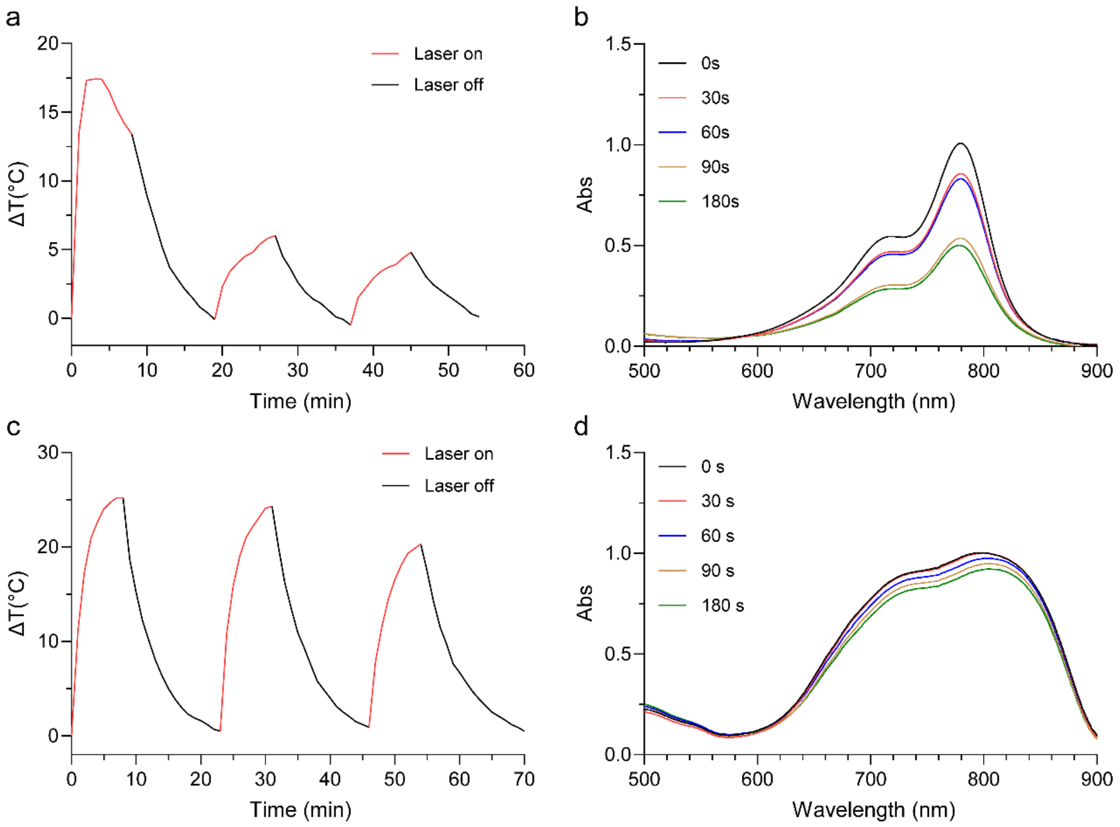

Figure S5. Photostability of free ICG and ICG@2%NGs in H_2O after multiple irradiations. a) Photothermal properties of free ICG in H_2O after multiple irradiations by 808 nm laser. b) UV-vis absorption of free ICG in H_2O after multiple irradiations. c) Photothermal properties of ICG@2%NGs in H_2O after multiple irradiations by 808 nm laser. d) UV-vis absorption of ICG@2%NGs in H_2O after multiple irradiations.

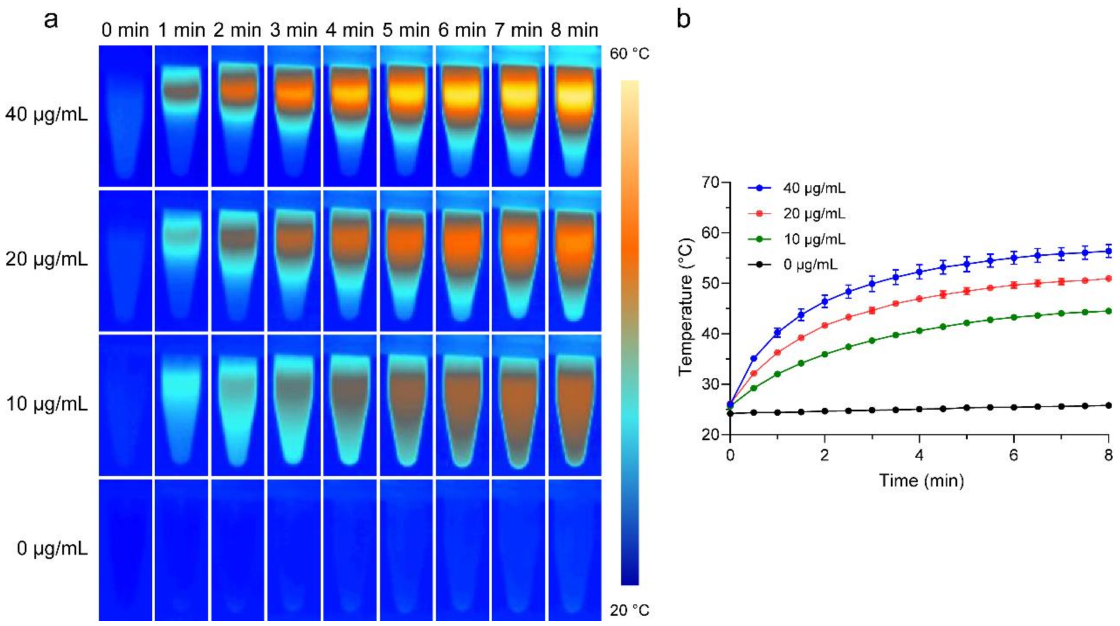

Figure S6. Concentration-dependent photothermal properties of ICG@2%NGs in H_2O. a) Infrared images of concentration-dependent photothermal properties of ICG@2%NGs in H_2O with 808 nm laser power of 1W/cm^2. b) Concentration-dependent photothermal properties of ICG@2%NGs in H_2O. Data are presented as mean values ± SD (n = 3 independent replicates).

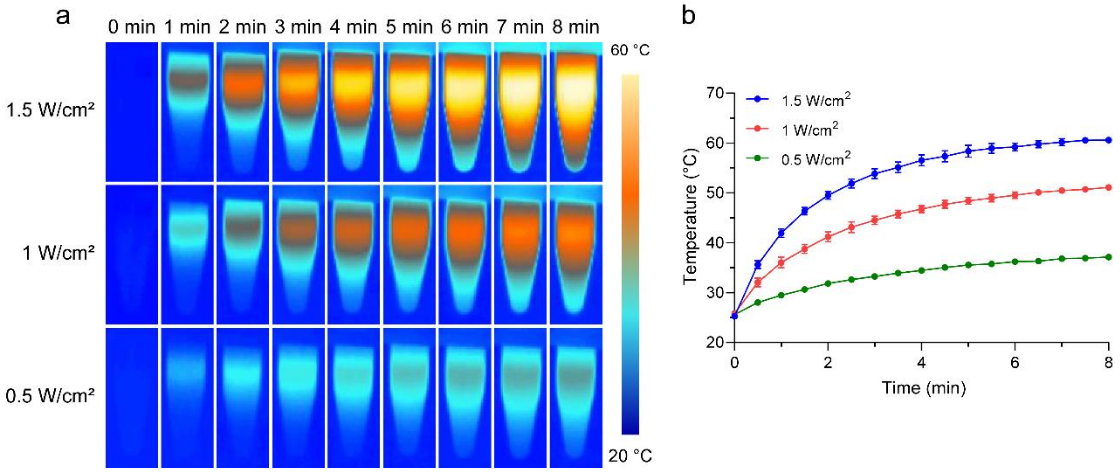

Figure S7. Power-dependent photothermal properties of ICG@2%NGs in H_2O. a) Infrared images of power-dependent photothermal properties of ICG@2%NGs in H_2O with ICG concentration of 20 μg/mL. b) Power-dependent photothermal properties of ICG@2%NGs in H_2O. Data are presented as mean values ± SD (n = 3 independent replicates).

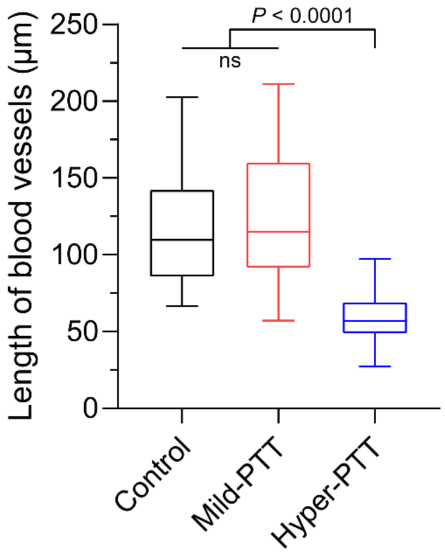

Figure S8. Length of blood vessels after mild-PTT or hyper-PTT. Box plots indicate median

(middle line), 25th, 75th percentile (box) and minimum and maximum (whiskers), (n = 40 independent replicates). Statistical significance was calculated by one-way ANOVA.

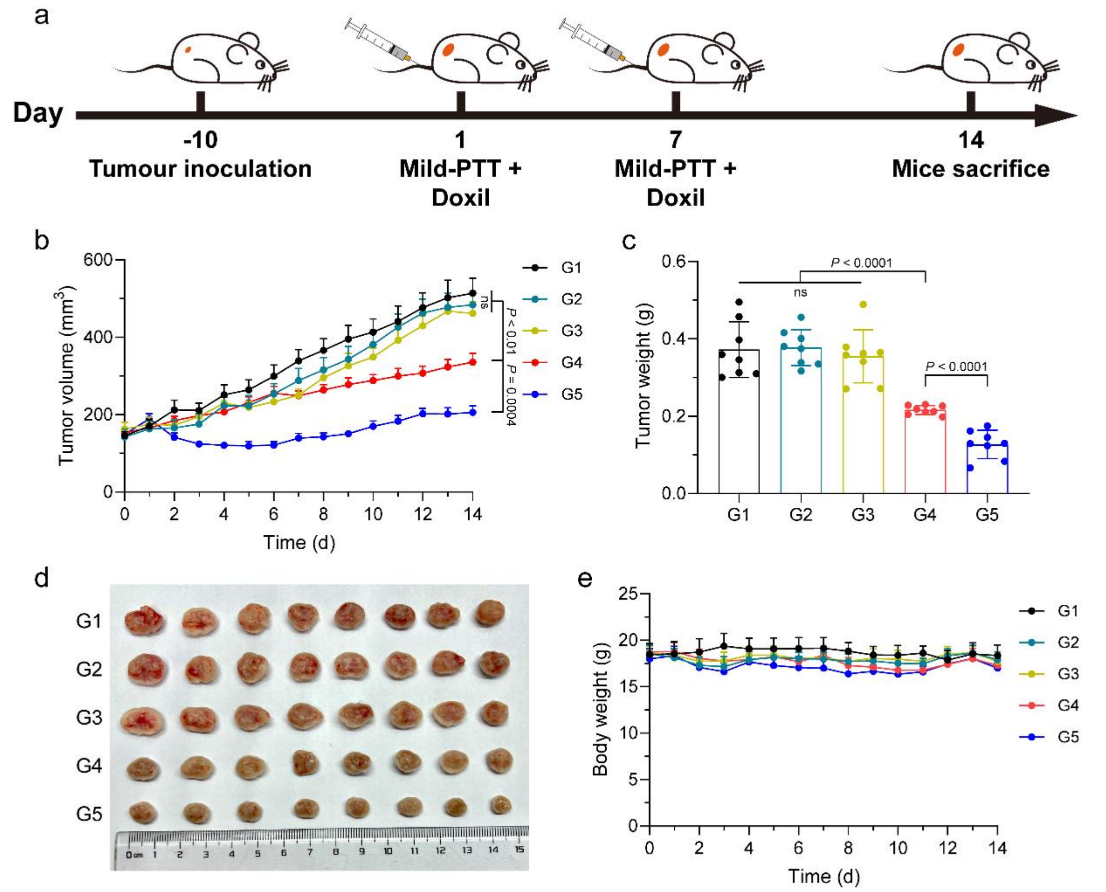

G1: Control G2: ICG@2%NGs G3: ICG@2%NGs (laser) G4: ICG@2%NGs + Doxil G5: ICG@2%NGs (laser) + Doxil

Figure S9. Antitumor efficacy of Doxil with mild-PTT-enhanced blood perfusion. a) Illustration of drug administration strategy. b) Tumor growth profiles of 4T1 tumor-bearing mice after treatment by Doxil with mild-PTT-enhanced blood perfusion by ICG@2%NGs. Data are presented as mean values ± SEM (n = 8 biological independent replicates). c) Weight of tumors after treatment. Data are presented as mean values ± SD (n = 8 biological independent replicates). d) Photograph of tumors harvested from the mice after treatment. e) Body weight profiles of mice after treatment. Data are presented as mean values ± SEM (n = 8 biological independent replicates). Statistical significance was calculated by unpaired two-sided Student's t-test. G1, Control; G2, ICG@2%NGs; G3, ICG@2%NGs (laser); G4, ICG@2%NGs + Doxil; G5, ICG@2%NGs (laser) + Doxil.

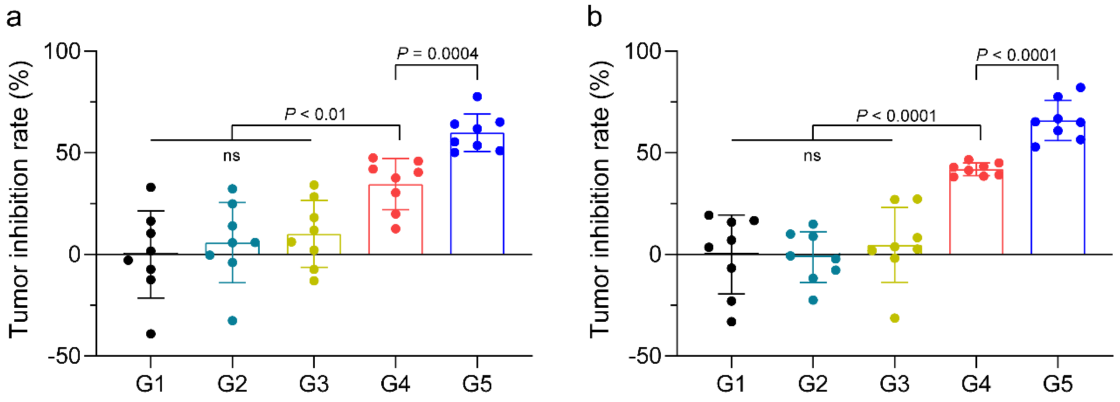

Figure S10. Tumor inhibition rate of Doxil with mild-PTT-enhanced blood perfusion. Volume-based (a) and weight-based (b) tumor inhibition rate. Data are presented as mean values ± SD (*n* = 8 biological independent replicates). Statistical significance was calculated by unpaired two-sided Student's t-test. G1, Control; G2, ICG@2%NGs; G3, ICG@2%NGs (laser); G4, ICG@2%NGs + Doxil; G5, ICG@2%NGs (laser) + Doxil.

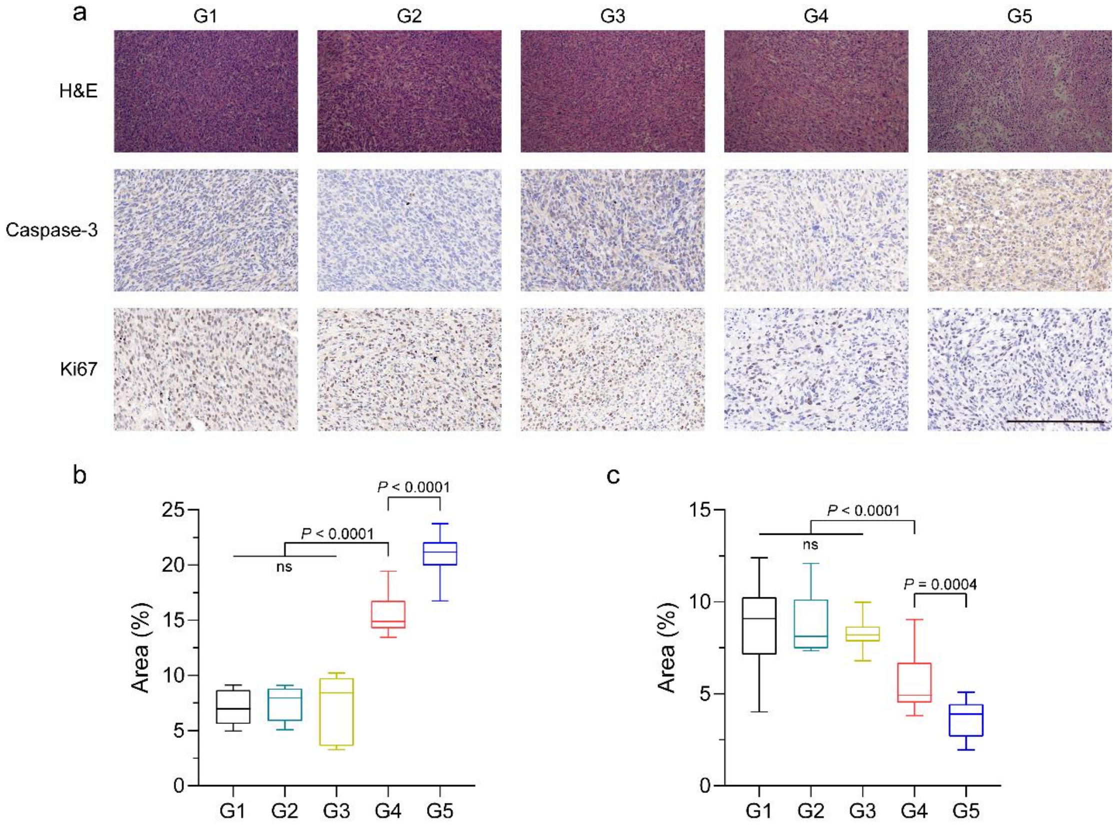

Figure S11. Apoptosis and proliferation of tumor cells after treatment by Doxil with mild-PTT-enhanced blood perfusion. a) H&E, Capase-3 and Ki67 staining of tumors. Scale bar = 500 μm. Percentage of the area of apoptotic cells (b) and proliferative cells (c). Box plots indicate median (middle line), 25th, 75th percentile (box) and minimum and maximum

(whiskers) (n = 15 independent replicates). Statistical significance was calculated by unpaired two-sided Student's t-test. G1, Control; G2, ICG@2%NGs; G3, ICG@2%NGs (laser); G4, ICG@2%NGs + Doxil; G5, ICG@2%NGs (laser) + Doxil.

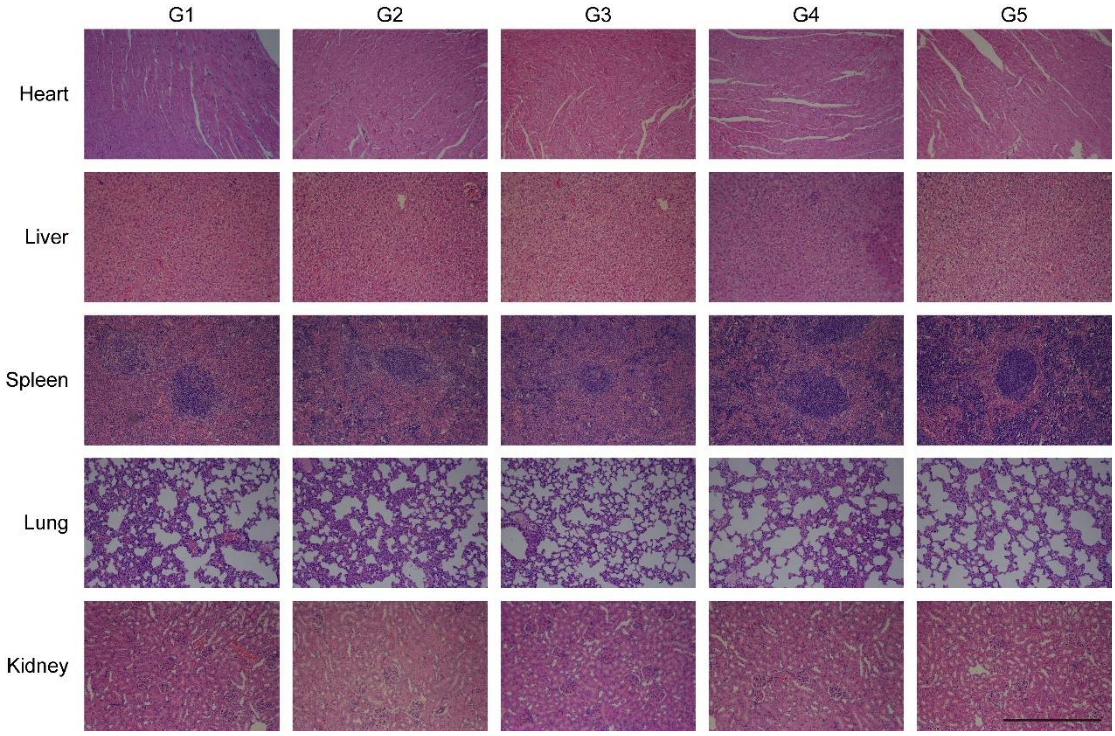

Figure S12. Treatment safety of Doxil with mild-PTT-enhanced blood perfusion. H&E staining of major organs, including heart, liver, spleen, lung and kidney. Scale bar = 500 μm. G1, Control; G2, ICG@2%NGs; G3, ICG@2%NGs (laser); G4, ICG@2%NGs + Doxil; G5, ICG@2%NGs (laser) + Doxil.

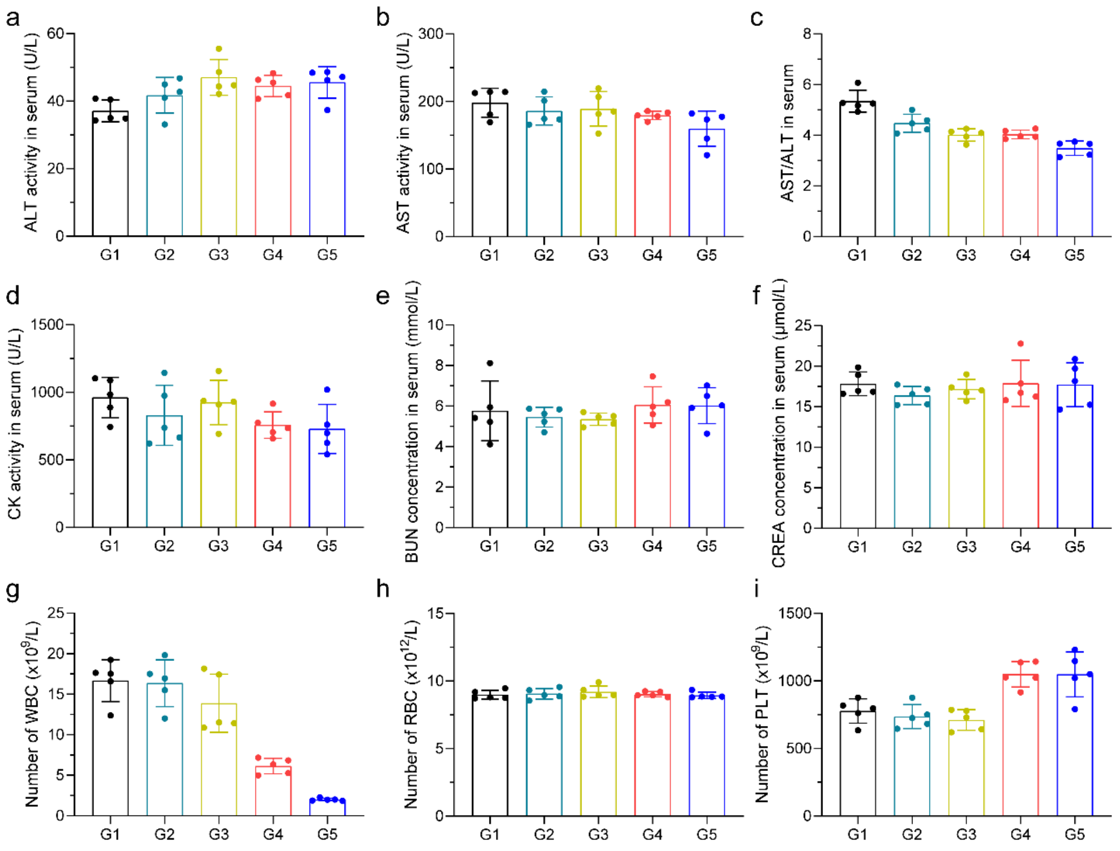

Figure S13. Treatment safety of Doxil with mild-PTT-enhanced blood perfusion. Blood biochemical analysis (a-f) and blood routine examine (g-i) after treatment. Data are presented as mean values ± SD (*n* = 5 biological independent replicates). G1, Control; G2, ICG@2%NGs; G3, ICG@2%NGs (laser); G4, ICG@2%NGs + Doxil; G5, ICG@2%NGs (laser) + Doxil.

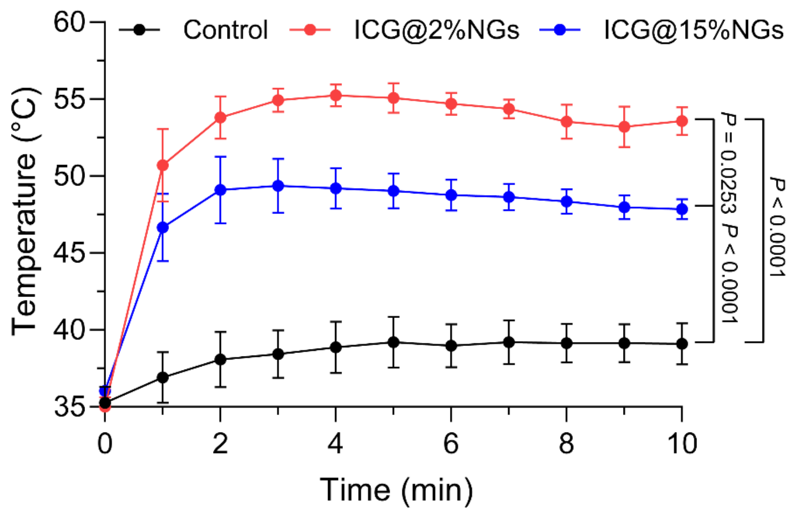

Figure S14. In vivo photothermal properties of ICG-loaded nanogels with different stiffness.

Data are presented as mean values ± SD (n = 3 biological independent replicates). Statistical significance was calculated by one-way ANOVA.

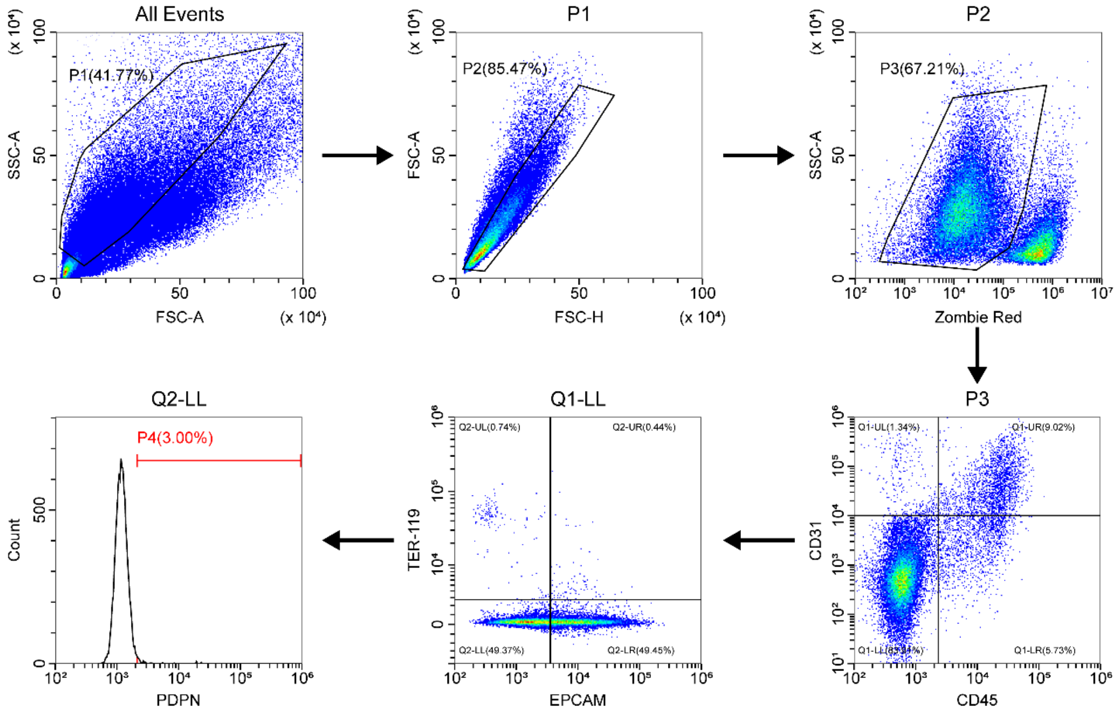

Figure S15. Gating strategy for identifying living CAFs in tumor tissues after mild-PTT by ICG-loaded nanogels with varied stiffness.

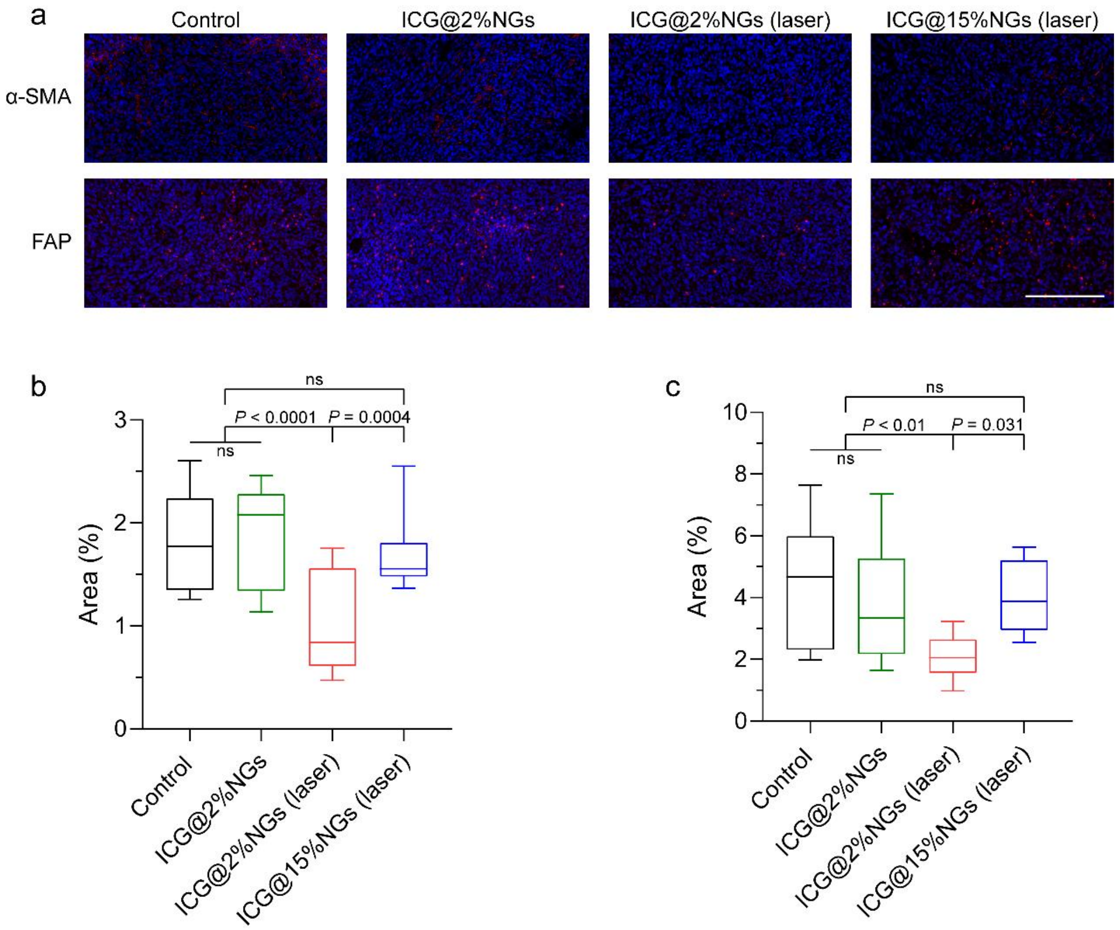

Figure S16. Fluorescent images of CAFs after mild-PTT by ICG-loaded nanogels with varied stiffness. a) α-SMA and FAP staining of tumors. Blue: nuclei of tumor cells. Red: CAFs. Scale bar = 200 μm. Percentage of the area of α-SMA-labeled (b) and FAP-labeled (c) CAFs. Box plots indicate median (middle line), 25th, 75th percentile (box) and minimum and maximum (whiskers) (n = 15 independent replicates). Statistical significance was calculated by one-way ANOVA.

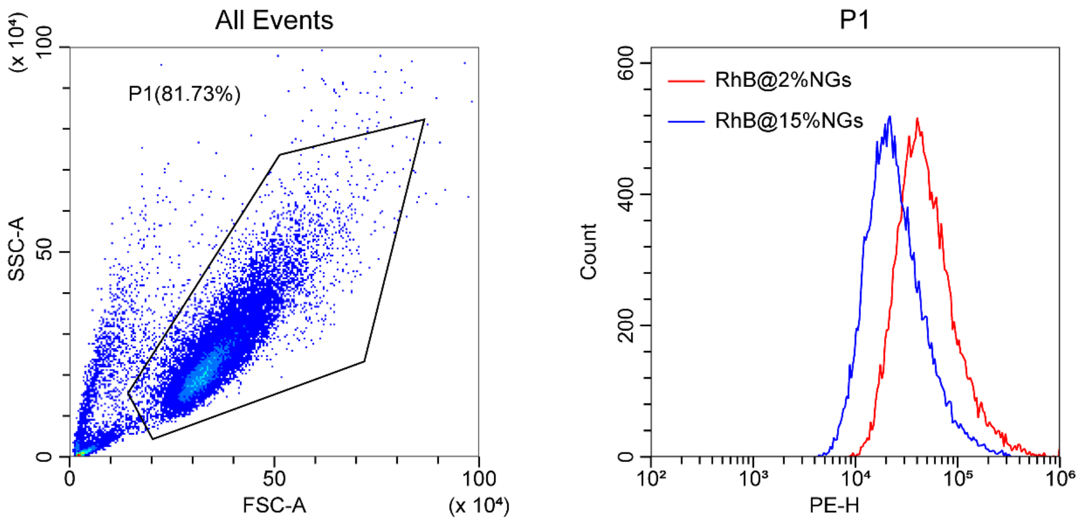

Figure S17. Gating strategy for cellular uptake of Rhodamine B-labeled nanogels with varied stiffness in CAFs.

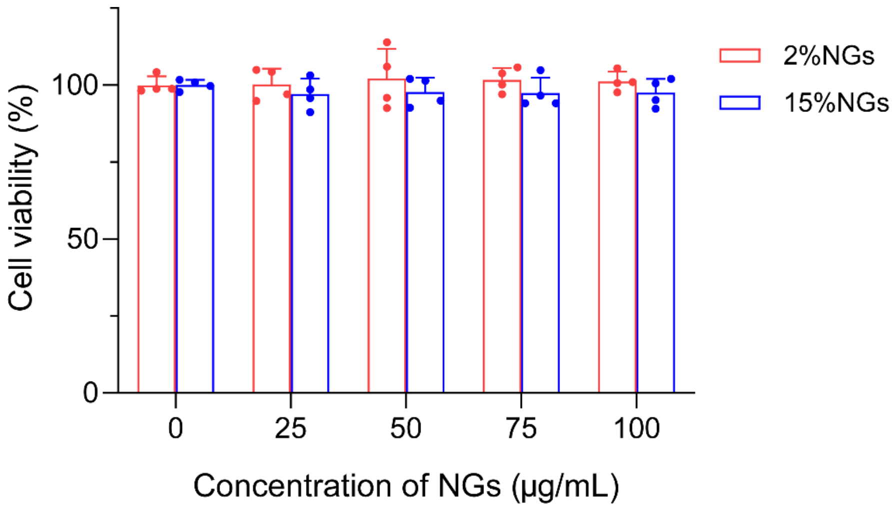

Figure S18. Cytotoxicity of nanogels with different stiffness for 24 h in CAFs. Data are presented as mean values ± SD (n = 4 biological independent replicates).

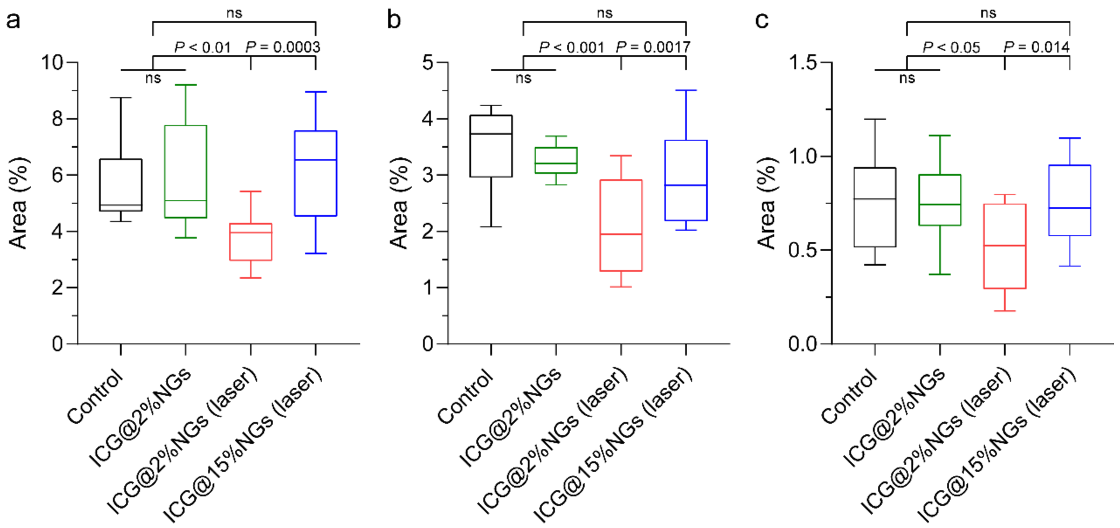

Figure S19. Percentage of ECM area after mild-PTT by ICG-loaded nanogels with different stiffness. Percentage of the area of total collagen (Masson) (a), collagen I (b) and fibronectin (c). Box plots indicate median (middle line), 25th, 75th percentile (box) and minimum and maximum (whiskers) (n = 15 independent replicates). Statistical significance was calculated by one-way ANOVA.

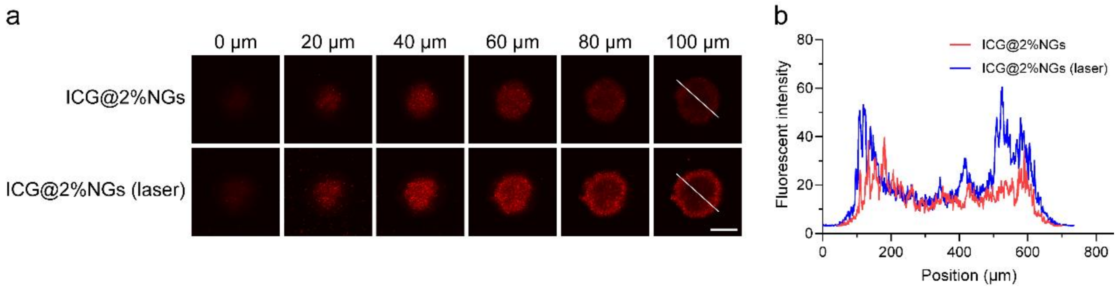

Figure S20. Penetration of soft RhB@2%NGs after mild-PTT by ICG@2%NGs in 3D tumor spheroids. a) Penetration of RhB@2%NGs after mild-PTT by ICG@2%NGs. Scale bar = 200 μm. b) Distribution of fluorescent intensity along radial direction in (a).

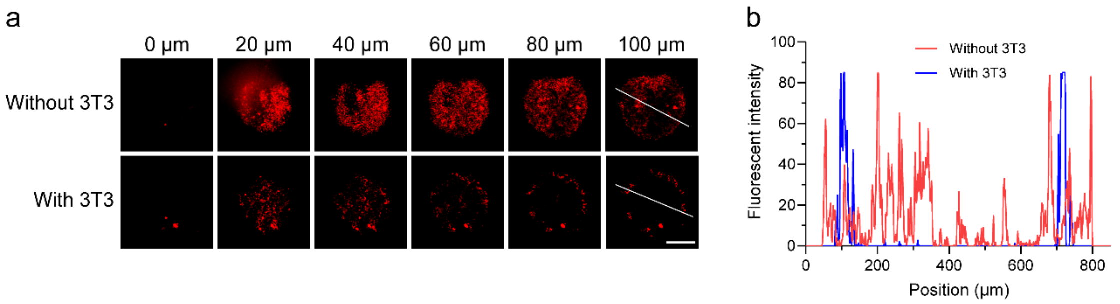

Figure S21. Penetration of stiff RhB@15%NGs in 3D tumor spheroids with or without

matrix. a) Penetration of RhB@15%NGs in 3D tumor spheroids with or without 3T3 cells. Scale bar = 200 μm. b) Distribution of fluorescent intensity along radial direction in (a).

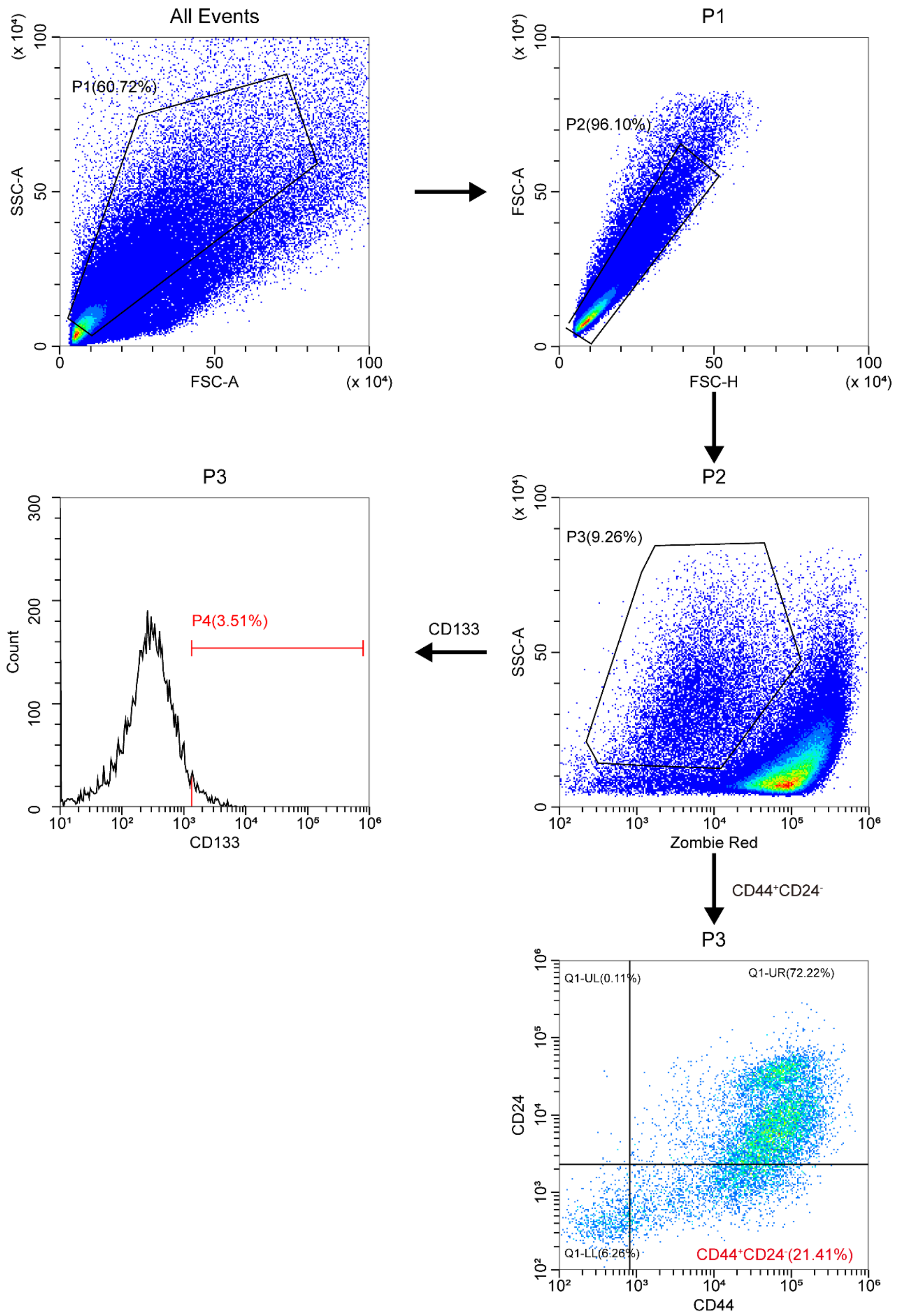

Figure S22. Gating strategy for identifying living CSCs in tumor tissues after mild-PTT by ICG-loaded nanogels with varied stiffness.

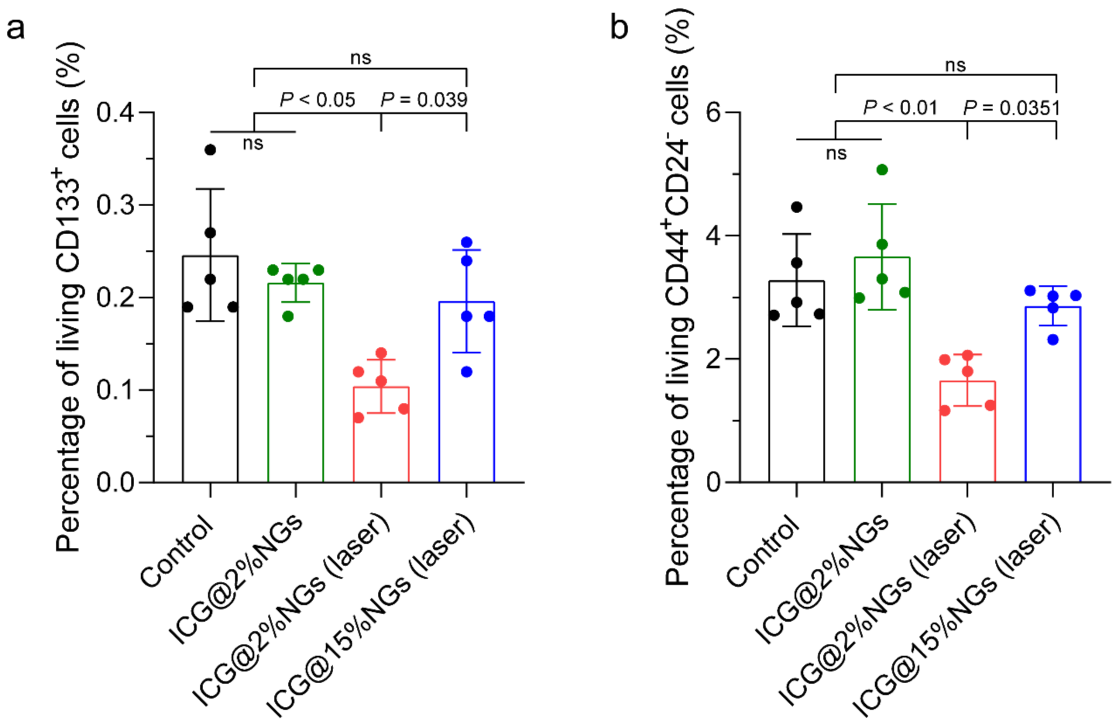

Figure S23. Percentage of living CSCs after mild-PTT by ICG-loaded nanogels with varied stiffness. Percentage of living $CD133^+$ cells (a) and $CD44^+CD24^-$ cells (b) after mild-PTT. Data are presented as mean values ± SD (n = 5 biological independent replicates). Statistical significance was calculated by one-way ANOVA.

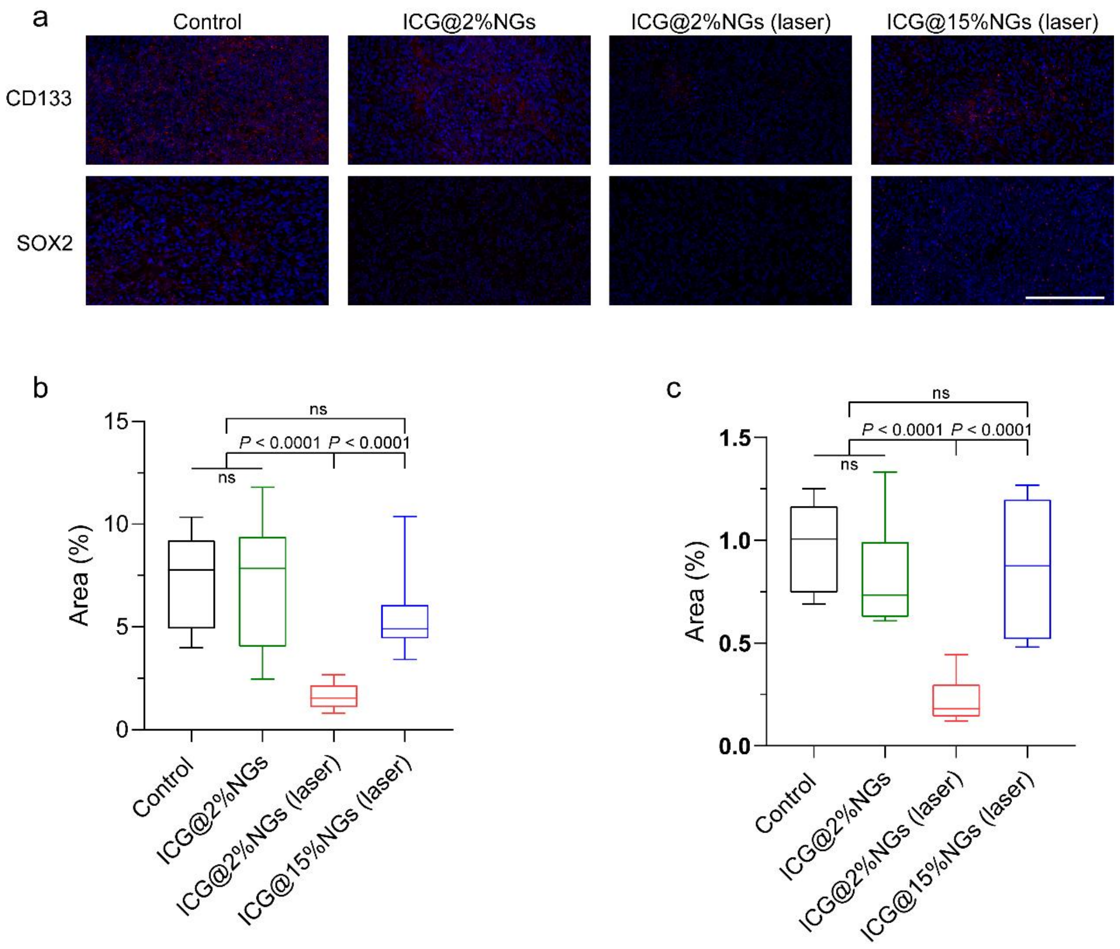

Figure S24. Fluorescent images of CSCs after mild-PTT by ICG-loaded nanogels with varied stiffness. a) CD133 and SOX2 staining of tumors. Blue: nuclei of tumor cells. Red: CSCs. Scale bar = 200 μm. Percentage of the area of CD133-labeled (b) and SOX2-labeled (c) CSCs. Box plots indicate median (middle line), 25th, 75th percentile (box) and minimum and maximum (whiskers) (n = 15 independent replicates). Statistical significance was calculated by one-way ANOVA.

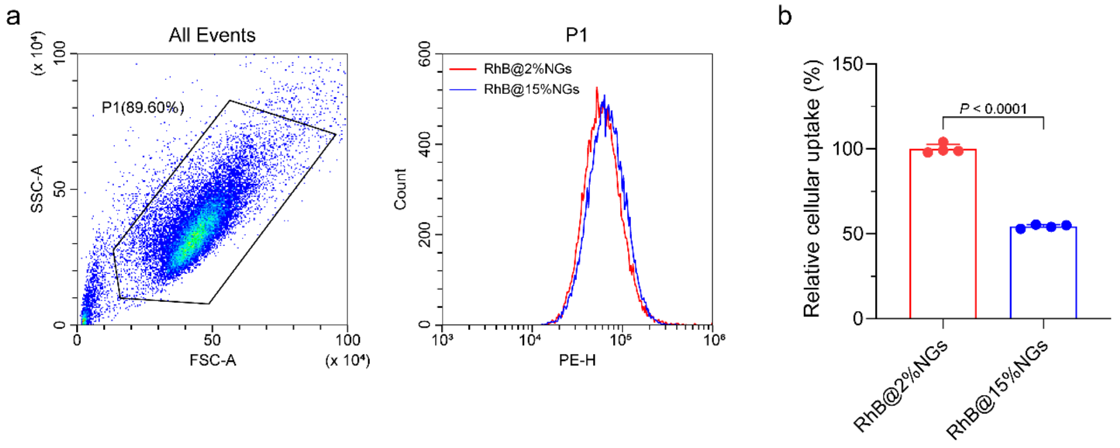

Figure S25. Relative cellular uptake of RhB-labeled nanogels of varied stiffness in CSCs. a) Gating strategy for cellular uptake of Rhodamine B-labeled nanogels with different stiffness in CSCs. b) Relative cellular uptake of RhB-labeled nanogels with different stiffness in CSCs. Data are presented as mean values ± SD (n = 4 biological independent replicates). Statistical significance was calculated by unpaired two-sided Student's t-test.

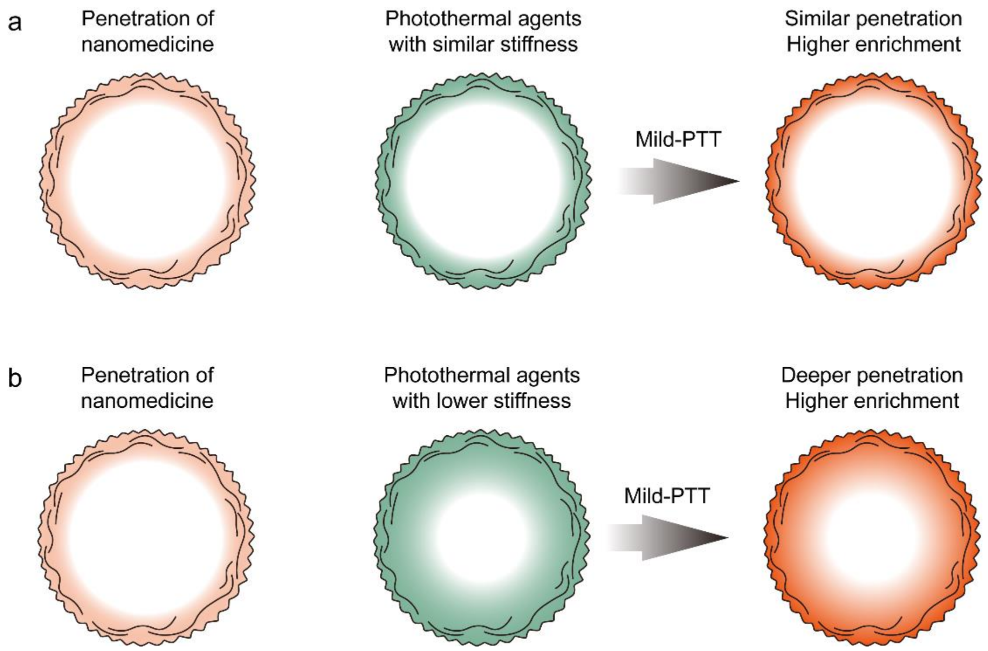

Figure S26. Illustration of nanomedicine penetration and enrichment affected by photothermal agents with varied stiffness. a) Penetration of nanomedicine affected by photothermal agents with similar stiffness. After mild-PTT mediated by photothermal agents with similar stiffness, nanomedicine exhibits higher enrichment but the penetration is similar to photothermal agents. b) Penetration of nanomedicine affected by photothermal agents with lower stiffness. After mild-PTT mediated by photothermal agents with lower stiffness, nanomedicine achieved higher enrichment and deeper penetration.

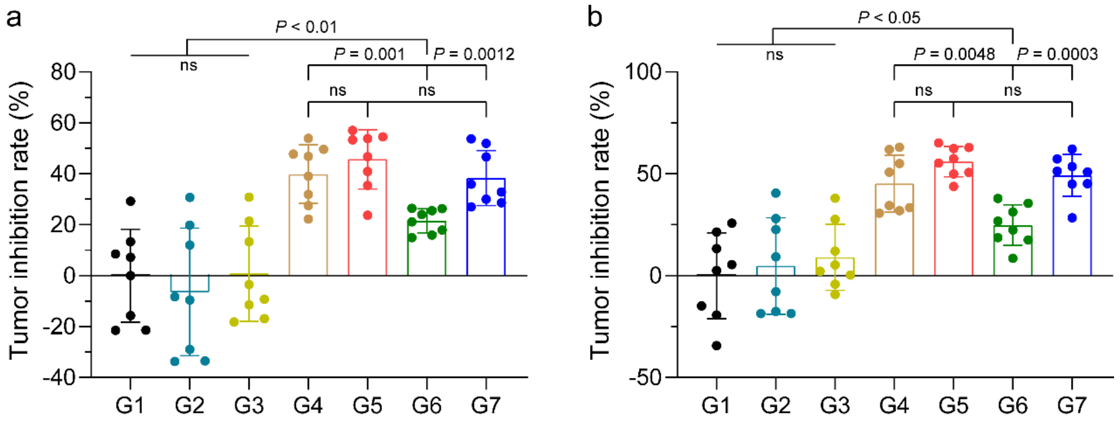

Figure S27. Tumor inhibition rate of DOX-loaded nanogels with different stiffness as promoted by ICG@2%NGs-mediated mid-PTT. Volume-based (a) and weight-based (b) tumor inhibition rate. Data are presented as mean values ± SD (n = 8 biological independent replicates). Statistical significance was calculated by unpaired two-sided Student's t-test. G1, Control; G2, ICG@2%NGs; G3, ICG@2%NGs (laser); G4, ICG@2%NGs + DOX@2%NGs; G5, ICG@2%NGs (laser) + DOX@2%NGs; G6, ICG@2%NGs + DOX@15%NGs; G7, ICG@2%NGs (laser) + DOX@15%NGs.

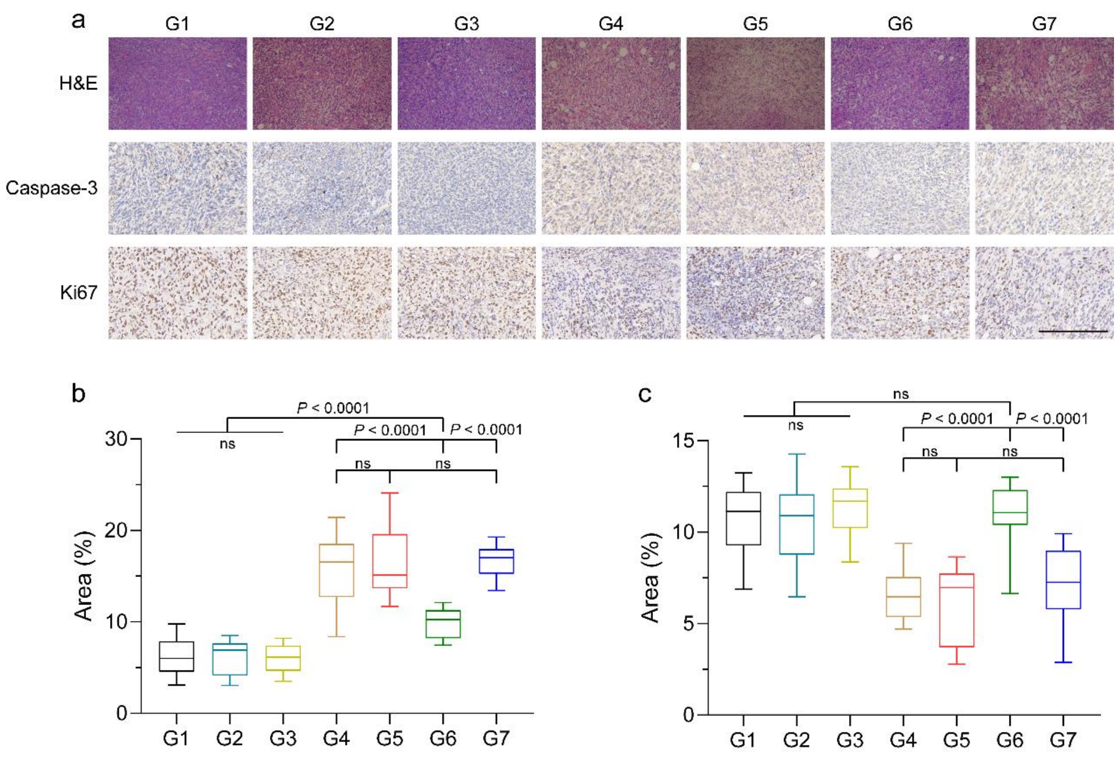

Figure S28. Apoptosis and proliferation of tumor cells after treatment by DOX-loaded nanogels with different stiffness as promoted by ICG@2%NGs-mediated mid-PTT. a) H&E,

Capase-3 and Ki67 staining of tumors. Scale bar = 500 μm. Percentage of the area of apoptotic cells (b) and proliferative cells (c). Box plots indicate median (middle line), 25th, 75th percentile (box) and minimum and maximum (whiskers) (*n* = 15 independent replicates). Statistical significance was calculated by unpaired two-sided Student's t-test. G1, Control; G2, ICG@2%NGs; G3, ICG@2%NGs (laser); G4, ICG@2%NGs + DOX@2%NGs; G5, ICG@2%NGs (laser) + DOX@2%NGs; G6, ICG@2%NGs + DOX@15%NGs; G7, ICG@2%NGs (laser) + DOX@15%NGs.

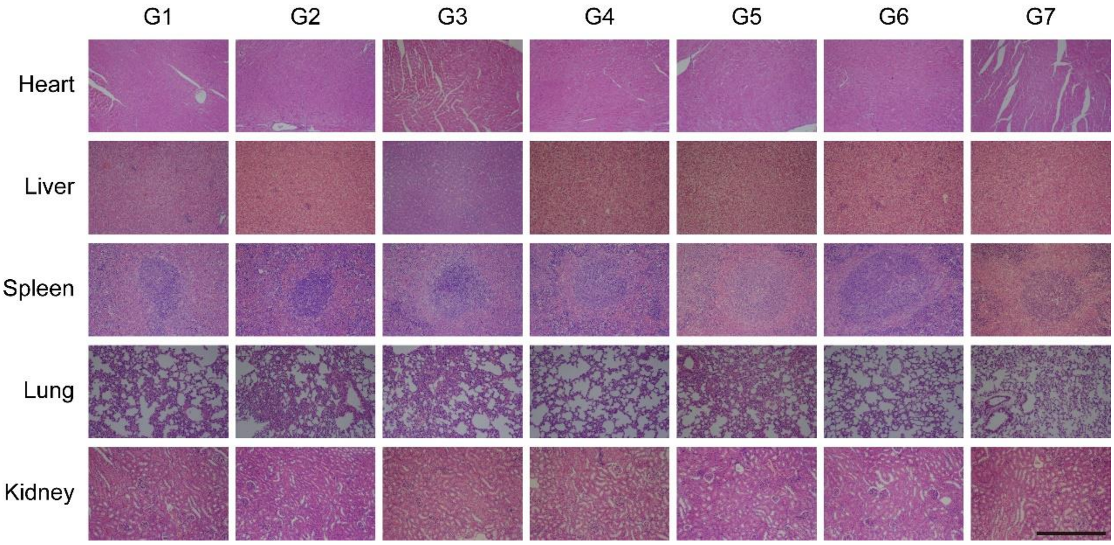

Figure S29. Treatment safety by DOX-loaded nanogels with different stiffness as promoted by ICG@2%NGs-mediated mid-PTT. H&E staining of major organs, including heart, liver, spleen, lung and kidney. Scale bar = 500 μm. G1, Control; G2, ICG@2%NGs; G3, ICG@2%NGs (laser); G4, ICG@2%NGs + DOX@2%NGs; G5, ICG@2%NGs (laser) + DOX@2%NGs; G6, ICG@2%NGs + DOX@15%NGs; G7, ICG@2%NGs (laser) + DOX@15%NGs.

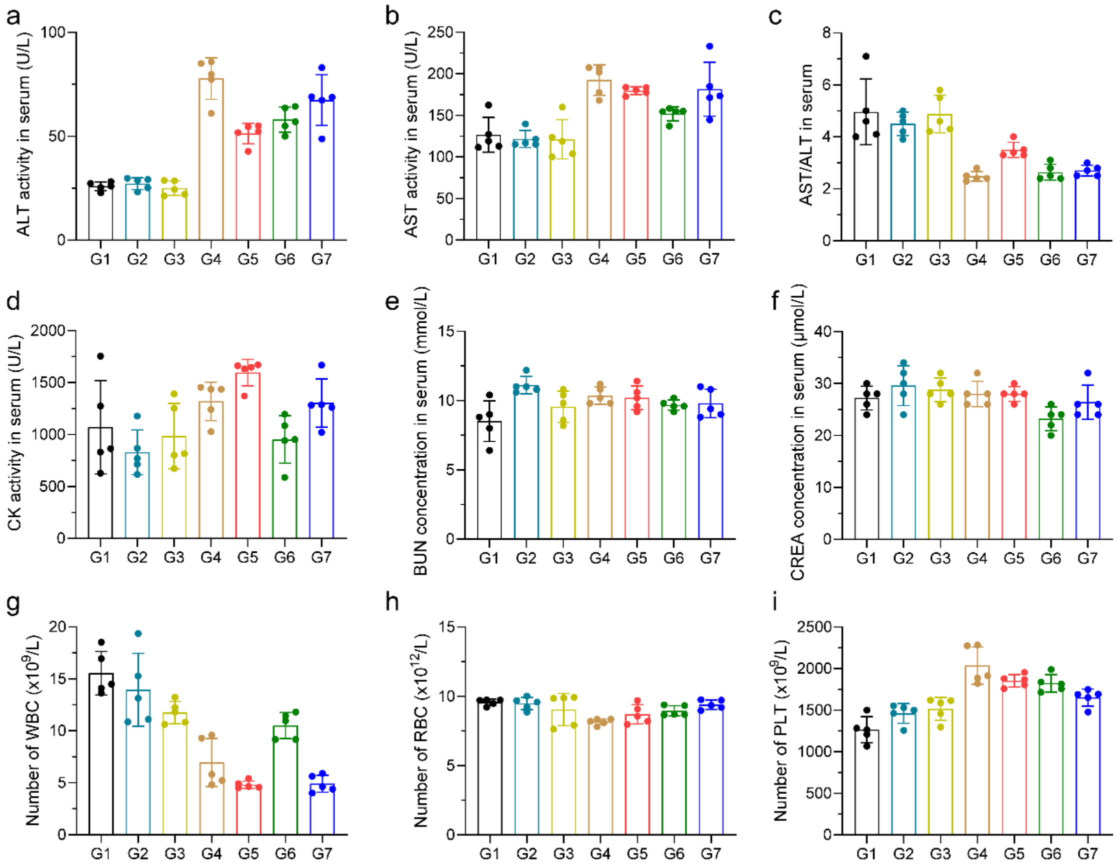

Figure S30. Treatment safety by DOX-loaded nanogels with different stiffness as promoted by ICG@2%NGs-mediated mid-PTT. Blood biochemical analysis (a-f) and blood routine examine (g-i) after treatment. Data are presented as mean values ± SD (*n* = 5 biological independent replicates). G1, Control; G2, ICG@2%NGs; G3, ICG@2%NGs (laser); G4, ICG@2%NGs + DOX@2%NGs; G5, ICG@2%NGs (laser) + DOX@2%NGs; G6, ICG@2%NGs + DOX@15%NGs; G7, ICG@2%NGs (laser) + DOX@15%NGs.

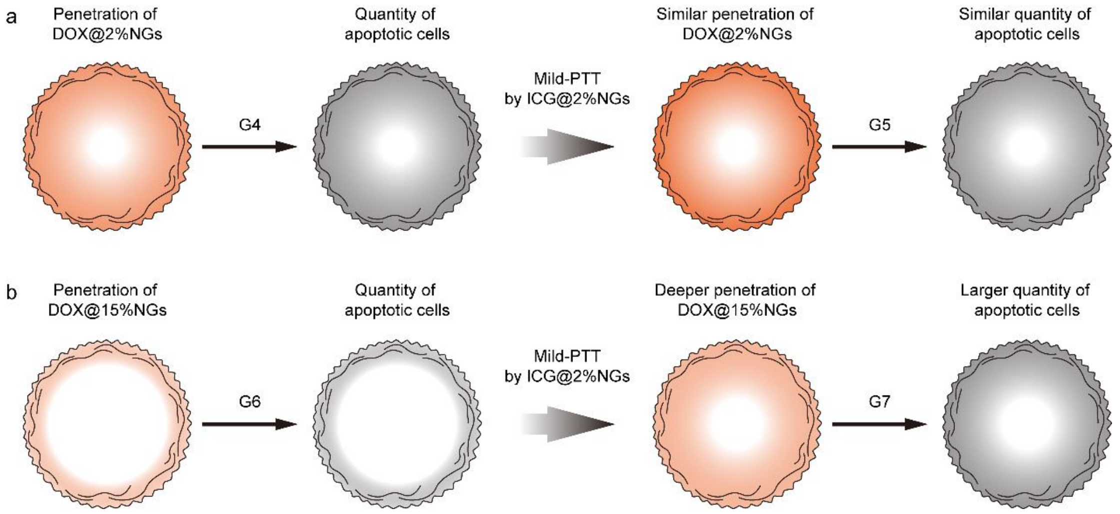

Figure S31. Illustration of nanomedicine with varied stiffness affected by soft photothermal agents. a) Tumor accumulation and antitumor efficacy of soft nanomedicine affected by soft photothermal agents. After mild-PTT mediated by ICG@2%NGs, DOX@2%NGs exhibits higher enrichment but the penetration has not been promoted. As a result, similar antitumor effects have been achieved in G4 and G5. b) Tumor accumulation and antitumor efficacy of stiff nanomedicine affected by soft photothermal agents. After mild-PTT mediated by ICG@2%NGs, the enrichment and penetration of stiff DOX@15%NGs has been promoted. Therefore, G7 achieves better antitumor effect than G6.
